# Mixed-methods non-randomised single-arm feasibility study assessing delivery of a remote vocational rehabilitation intervention for patients with serious injury: the ROWTATE study

**DOI:** 10.1136/bmjopen-2025-104518

**Published:** 2025-11-27

**Authors:** Blerina Kellezi, Jain Holmes, Jade Kettlewell, Rebecca Lindley, Kate Radford, Priya Patel, Kay Bridger, Natasha A Lannin, Isobel Andrews, Lauren Blackburn, Adam Brooks, Roshan das Nair, Steve Fallon, Amanda Farrin, K Hoffman, Trevor Jones, Richard Morriss, Stephen Timmons, Denise Kendrick

**Affiliations:** 1 Department of Psychology, Nottingham Trent University, Nottingham, UK; 2 Centre for Academic Primary Care, School of Medicine, University of Nottingham, Nottingham, UK; 3 Centre for Rehabilitation & Ageing Research, University of Nottingham, Nottingham, UK; 4 Mental Health and Clinical Neuroscience, University of Nottingham, Nottingham, UK; 5 Centre for Rehabilitation and Ageing, University of Nottingham, Nottingham, UK; 6 NIHR Nottingham Biomedical Research Centre, Nottingham, UK; 7 Institute of Mental Health, University of Nottingham School of Medicine, Nottingham, UK; 8 Neuroscience, Monash University Faculty of Medicine Nursing and Health Sciences, Melbourne, Victoria, Australia; 9 Department of Major Trauma, East Midlands Major Trauma Centre, Queen’s Medical Centre, Nottingham, UK; 10 SINTEF, Trondheim, Norway; 11 Clinical Trials Research Unit, University of Leeds, Leeds, UK; 12 Queen Mary University of London, London, UK; 13 Centre for Health Innovation, Leadership & Learning, University of Nottingham, Nottingham University Business School, Nottingham, UK

**Keywords:** Trauma, REHABILITATION MEDICINE, Psychological Stress, Feasibility Studies, QUALITATIVE RESEARCH, EDUCATION & TRAINING (see Medical Education & Training)

## Abstract

**Objectives:**

This study aimed to evaluate the feasibility of delivering a vocational rehabilitation intervention (Return to Work After Trauma—ROWTATE), remotely to individuals recovering from traumatic injuries. The primary objectives were to assess therapists’ training and competence, adapt the intervention and training for remote delivery and assess the feasibility and fidelity of remote delivery to inform a definitive randomised controlled trial.

**Design:**

A mixed-methods feasibility study incorporating (1) telerehabilitation qualitative literature review, (2) qualitative interviews preintervention and postintervention with therapists and patients, (3) a team objective structured clinical examination to assess competency, (4) usefulness of training, attitudes towards (15-item Evidence-Based Practice Attitude Scale) and confidence in (4-item Evidence Based Practice Confidence Scale) evidence-based practice, intervention delivery confidence (8-bespoke questions) and intervention behaviour determinants (51-items Theoretical Domains Framework) and (5) single-arm intervention delivery feasibility study.

**Setting:**

The study was conducted in two UK Major Trauma Centres. The intervention and training were adapted for remote delivery due to the COVID-19 pandemic.

**Participants:**

Therapists: Seven occupational therapists (OTs) and clinical psychologists (CPs) were trained, and six participated in competency assessment. Seven OTs and CPs participated in preintervention interviews and surveys; six completed post-intervention interviews and four completed post-training surveys. Patients: 10 patients were enrolled in the single-arm feasibility study and 4 of these participated in postintervention qualitative interviews. Inclusion criteria included therapists involved in vocational rehabilitation delivery and patients admitted to major trauma centres. Exclusion criteria included participation in other vocational rehabilitation trials or those who had returned to work or education for at least 80% of preinjury hours. Intervention: The ROWTATE vocational rehabilitation intervention was delivered remotely by trained OTs and CPs. Training included competency assessments, mentoring and adaptation for telerehabilitation. The intervention was delivered over multiple sessions, with content tailored to individual patient needs.

**Results:**

Therapists found the training useful, reported positive attitudes (Evidence-Based Practice Attitude Scale mean=2.9 (SD 0.9)) and high levels of confidence in delivering evidence-based practice (range 75%–100%) and the ROWTATE intervention (range 80%–100%). Intervention barriers identified pretraining became facilitators post-training. Half the therapists needed additional support post-training through mentoring or additional training. The intervention and training were successfully adapted for remote delivery. High levels of fidelity (intervention components delivered: OTs=84.5%, CPs=92.9%) and session attendance rates were found (median: OT=97%, CP=100%). Virtually all sessions were delivered remotely (OT=98%, CP=100%). The intervention was acceptable to patients and therapists; both considered face-to-face delivery where necessary was important.

**Conclusions:**

The ROWTATE intervention was delivered remotely with high fidelity and attendance and was acceptable to patients and therapists. Definitive trial key changes include modifying therapist training, competency assessment, face-to-face intervention delivery where necessary and addressing lower fidelity intervention components.

**Trial registration number:**

ISRCTN74668529.

**STRENGTHS AND LIMITATIONS OF THIS STUDY**

- The development and delivery of the Return to Work After Trauma training included patient perspectives to enhance relevance for therapists.
- Training occupational therapists and clinical psychologists together helped build strong working relationships.
- Consideration of local issues and barriers helped tailor the intervention to specific needs.
- The study successfully recruited a diverse group of patients during the pandemic, providing valuable experience for a definitive trial. However, recruitment during the pandemic may not reflect recruitment beyond the pandemic and limited assessment of retention.
- The small number of therapists and patients limited the ability to statistically assess training effectiveness and intervention fidelity.

## Background

 Injuries are very common and remain one of the leading causes of death among working-age adults worldwide^1^ and in the UK.^2^ Injuries lead to significant use of health services; in 2020/2021 alone, there were over 367 000 hospital admissions in England among working-age adults.^3^ The introduction of major trauma centres has improved injury survival rates^4^; however, many occupational^5^ and psychological needs^6^ remain unmet. Injuries can lead to long-lasting physical^7^,^9^ and psychological effects,^10 11^ difficulties returning to work^12^ and education^13^,^16^ and reduced work productivity.

Vocational rehabilitation interventions have been shown to improve return to work^17^ and employer outcomes^18^ in specific patient populations. Vocational rehabilitation is defined as a multiple-professional approach to optimise participation in work among working-age individuals facing health-based work barriers and limitations.^19^ Evidence^20 21^ of effectiveness in general injury populations is limited; however, especially in relation to the provision of psychological support within vocational rehabilitation interventions.^20^,^24^ To address this evidence gap, as part of a programme of research (rowtate.org.uk), we developed an intervention to enhance Return to Work After Trauma (the ROWTATE intervention),^25^ which aims to address both vocational rehabilitation and psychological needs. The ROWTATE intervention is aimed at improving return to work and quality of life in working-age adults with at least moderate trauma (Injury Severity Score >8). The intervention involves an occupational therapist (OT) working in a case-coordination role with a wider team of healthcare professionals, employers, family members and other stakeholders (eg, solicitors, insurance agencies). The OT assesses the impact of the injury on the patient and their family, educates patients, employers and families about the impact of injury on work, monitors the patient’s postinjury goals, prepares patients for work, liaises with employers, the healthcare teams, other stakeholders as well as planning and monitoring a phased return to work. Patients are screened by the OT for mental health problems using standardised assessments at the initial session and 6 months later. Patients meeting predefined thresholds on any of the measures are discussed with or referred to a clinical psychologist (CP) for assessment and support. OTs and CPs receive monthly mentoring from an experienced OT or CP. The intervention commences within 12 weeks of injury and is tailored in duration and frequency according to individual need over a 12-month period (for more details, see the published protocol and ISRCTN registration #ISRCTN74668529).^25^

This paper presents findings from a mixed-methods study assessing the feasibility of delivering the ROWTATE intervention to inform development and delivery of a definitive randomised controlled trial (RCT), within the 6-year ROWTATE research programme (https://www.rowtate.org.uk). The study took place during the COVID-19 pandemic (thereafter pandemic). As a result, the intervention was adapted to be delivered and evaluated remotely, to ensure it could be delivered during the pandemic and beyond, as we anticipated that health services would continue to use remote interventions postpandemic.^26^ Systematic reviews have shown that telerehabilitation is as effective a delivery mode as face-to-face for some types of injuries^27 28^ or even superior to in-person rehabilitation.^27 29^ It has been found to be significantly cheaper per patient to deliver,^30^ to improve accessibility, be more convenient, enhance user autonomy and provide information about home circumstances,^31^ improve access to specialist services^32^ and reduce patient travel time^33^ and expenses.^33^ Remote delivery can also address the limited access to services^34 35^ that disproportionately affects those living in rural areas or a long distance from major trauma centres,^31 32 34^ although this does not overcome challenges faced by those who have limited access to the required technology.^36 37^ Recent systematic reviews have identified adequate training to be essential to enhance competency in health professionals and overcome challenges of remote healthcare delivery,^38^ including evidence among OTs.^39^ The importance of remote training for healthcare professionals has also been highlighted.^40^

The original feasibility study objectives were also adapted as a result of the pandemic. This resulted in being unable to assess recruitment strategies, follow-up data collection tools and processes, follow-up data completeness and retention rates. However, the definitive trial includes an internal pilot, so this will allow assessment of these factors. Adapting the original study objectives also resulted in not being able to evaluate importance and acceptability of outcome measures. As the outcome measures for the definitive trial were chosen based on extensive work with patient and public involvement representatives and a focus group of trauma patients,^41^ we considered further assessment of outcome measures was less important than other adaptations required to respond to the pandemic including producing an intervention which could be delivered during the pandemic and beyond. This paper will address one original study objective (objective 1) and three adapted objectives (objectives 2–4). These objectives were to (1) evaluate the OT and CP training, (2) adapt the OT and CP training to make it suitable for remote delivery, (3) adapt the ROWTATE intervention to make it suitable for remote delivery, via telerehabilitation and telepsychology and (4) deliver the adapted ROWTATE intervention and assess feasibility and fidelity of remote delivery via telerehabilitation. The final adapted objective (objective 5, to assess acceptability, barriers and facilitators to remote delivery of the ROWTATE intervention via telerehabilitation) has been addressed in detail in another publication.^42^ This study has been reported in accordance with the Consolidated Standards of Reporting Trials 2010 statement: extension to randomised pilot and feasibility trials.^43^

## Methodology

### Study design

**Figure 1:**
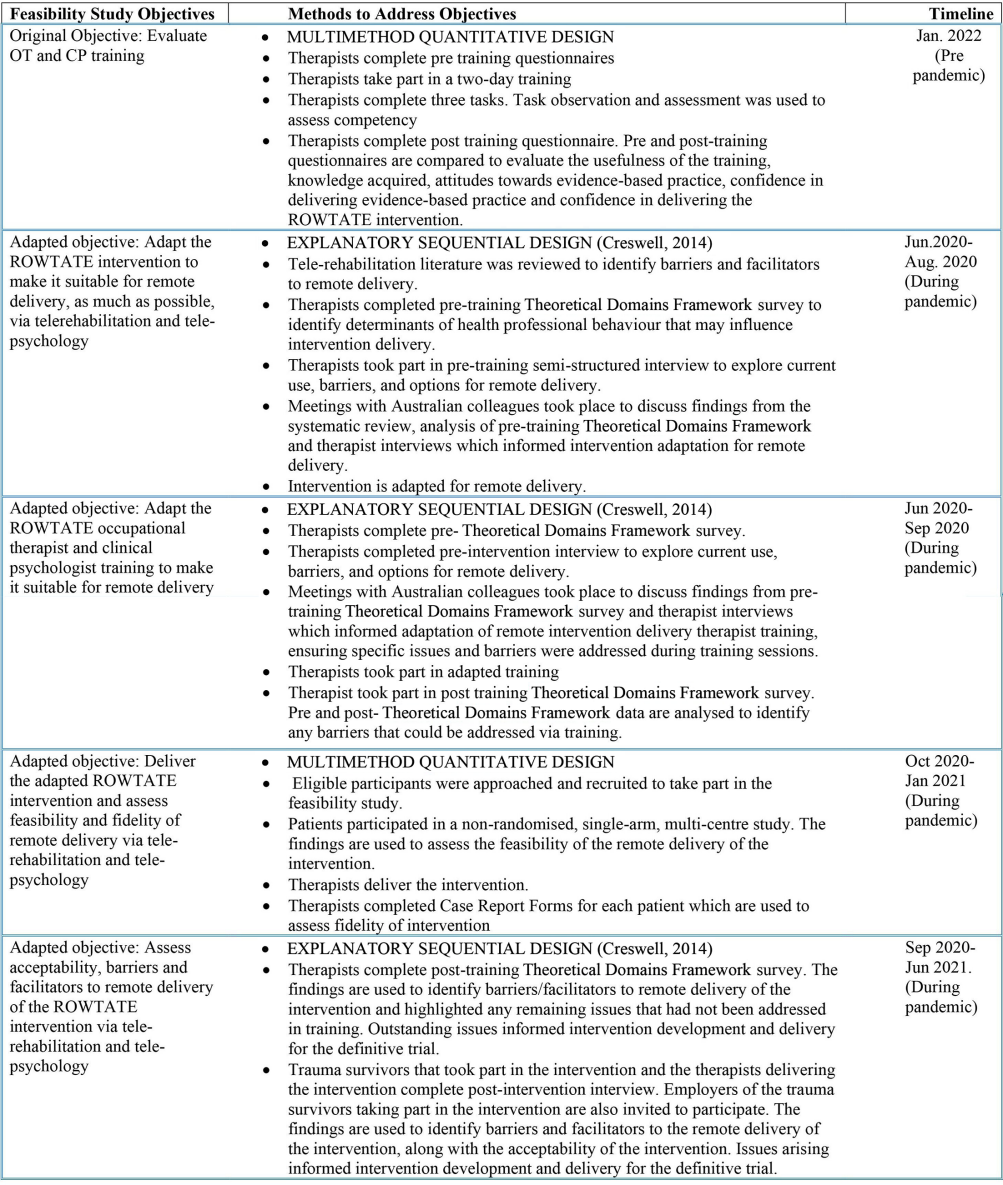
Objectives, methods and timeline The figure outlines the progression of objectives, methodological approaches and corresponding timelines for the feasibility study of the ROWTATE intervention. The study adapted its objectives in response to the COVID-19 pandemic to enable remote delivery of the ROWTATE intervention for traumatic injury patients combining occupational rehabilitation and clinical psychological support where relevant. Each objective is paired with a detailed methodological framework described using Creswell’s mixed-methods design, including surveys, interviews, training sessions and intervention delivery. CP, clinical psychologist; OT, occupational therapist; ROWTATE, Return to Work After Trauma.

Mixed-methods including (1) therapists’ competency assessment, (2) qualitative review of telerehabilitation literature, (3) intervention and training adaptation, (4) interviews with recruited patients and therapists (pre and post intervention), (5) surveys with therapists (pretraining and post-training) and (6) a single-arm intervention delivery feasibility study, were used to address the study objectives. The study uses different types of mixed methods for each objective (see figure 1). A mixed-methods approach was essential to capture both the quantitative outcomes (eg, attitude and confidence scores, fidelity, attendance) and qualitative insights (eg, therapist and patient experiences, barriers and facilitators) to inform the design of a definitive RCT. The study comprised four components (see figure 1 for a timeline of each methodology based on Creswell’s structure,^44^ and table 1 for GRAMMS checklist^45^ for reporting mixed method studies):

**Table 1:** Good Reporting of A Mixed Methods Study (GRAMMS) checklist for the ROWTATE mixed-methods study

Good Reporting of A Mixed Methods Study (GRAMMS) criteria	Study description
Justification for using a mixed-methods approach	Page 7 A mixed-methods approach was essential to capture both the quantitative outcomes (eg, fidelity, attendance, confidence scores) and qualitative insights (eg, therapist and patient experiences, barriers and facilitators) to inform the design of a definitive RCT.
Description of the design in terms of the purpose, priority and sequence of methods	The study used a different design for each objective. These are outlined in table 2.
Description of each method in terms of sampling, data collection and analysis	The study methods are described in detail in pages 7–21.
Description of where integration has occurred, how it has occurred and who has participated in it	Pages 7–11 and figure 1 Integration occurred during intervention adaptation, training development and interpretation of results. Qualitative findings explained quantitative outcomes. Integration was led by a multidisciplinary team including academics and practitioners from a range of disciplines (OT, psychology, general practice, psychiatry, trauma surgery, qualitative and implementation research, complex trials methodology, statistics and research management) and patient and public involvement.
Description of any limitation of one method associated with the presence of the other method	Pages 36–39 Small sample size limited statistical power for quantitative comparisons. Pandemic constraints affected recruitment and delivery.
Description of insights gained from mixing or integrating methods	Pages 36–39 Integration revealed that training mitigated initial barriers to remote delivery. Quantitative data confirmed high fidelity and confidence, while qualitative data explained how training adaptations and peer support for therapists contributed to these outcomes.

OT, occupational therapist; RCT, randomised controlled trial; ROWTATE, Return to Work After Trauma.

**Table 2:** Characteristics of therapists participating in the ROWTATE feasibility study

	OT	CP
Profession	4 (66.7%)	2 (33.3%)
Gender		
Female	4 (66.7%)	1 (16.7%)
Male	0	1 (16.7%)
National health service band		
7	3 (50.0%)	1 (16.7%)
8a	1 (16.7%)	1 (16.7%)
Place of training		
UK	3 (50.0%)	1 (16.7%)
International	1 (16.7%)	
Missing		1 (16.7%)
Years since qualification	Median=23 IQR=15, 28	Median=7.5 IQR=N/A
Had trauma rehabilitation experience		
Yes	4 (100.0%)	N/A
No	0 (0.0%)	
Length of trauma rehabilitation experience in years	Median=15 IQR=11, 17	N/A
Vocational rehabilitation experiences		
Yes	3 (75.0%)	N/A
No	1 (25.0%)	
Length of vocational rehabilitation experience in years	Median=8.5 IQR=3.5, 10	N/A
In the last 5 years has had at least 2 years’ experience of working with trauma patients		
Yes	N/A	1 (50.0%)
No		0 (0.0%)
Missing		1 (50.0%)

CP, clinical psychologist; N/A, not available; OT, occupational therapist; ROWTATE, Return to Work After Trauma.

### Initial training (objective 1: prepandemic)

The ROWTATE intervention was initially designed to be delivered face-to-face. Prepandemic, all OTs and CPs (hereafter referred to as therapists) participating in the study attended a 2-day interactive team workshop to provide them with the knowledge, skills and confidence to deliver the intervention. The training was based on similar training for OTs to deliver a vocational rehabilitation intervention for people with traumatic brain injury which has been shown to increase knowledge required for intervention delivery and be acceptable to therapists.^46^ The training, which was based on adult learning theory,^47^ used a mixture of didactic teaching, case vignette discussion and role play to achieve learning.^48^

### Patient and public involvement in training

The training involved patient and public involvement participants who interacted with the therapists and answered any questions they had. The training was evaluated by (1) comparing pretraining and post-training questionnaires and (2) conducting therapist competency assessment at the end of the workshop.

### Adapting the training and intervention in response to the pandemic (objectives 2 and 3: during pandemic)

After initial therapist training, the study objectives were adapted to enable remote delivery of the intervention and training. There were five stages to adapting the therapist training and intervention:

#### Literature review

The qualitative telerehabilitation literature was reviewed to identify barriers and facilitators to delivery.

#### Preadaptation training questionnaire

Therapists completed a questionnaire collecting information on determinants of health and professional behaviour that may influence intervention delivery.

#### Preintervention interviews

All recruited therapists participated in preintervention interviews to identify barriers and facilitators to telerehabilitation delivery.

#### Adapting the training and intervention

Therapist training and the intervention were adapted for remote delivery in a series of meetings by a team of four professionals (KR, JH, JK and NAL) using findings, shared learning and resources (including videos, case examples, work hardening packages, etc) from remote rehabilitation intervention trials in Australia, the literature review, pre-adapted training questionnaire and preintervention interviews. The adapted training was delivered remotely in September 2020. Refresher training was delivered remotely in June 2021.

#### Postadaptation training questionnaire

A postadapted training questionnaire administered within 1 month of the training collected the same information as the preadapted training questionnaire.

### Assessing feasibility of remote intervention delivery (objective 4: during pandemic)

The feasibility of delivering the remote intervention was assessed through a non-randomised, single-arm, multicentre study which included an assessment of the fidelity of intervention delivery.

### Postintervention interviews (objective 5: during pandemic)

Postintervention interviews were conducted with therapists and patients who received the intervention exploring acceptability of the intervention and issues regarding intervention delivery, including usefulness of the training. All patients recruited in the intervention and all trained therapists were invited to take part in the interview and those who agreed to take part completed the interview.

### Progression criteria

Criteria for progression to the main trial include remote delivery of the intervention (≥60%), OTs and CPs feeling the training prepared them sufficiently to deliver the remote intervention (≥70%), participants (≥90%) commencing the intervention within the specified time period (ie, 0–12 weeks postinjury), intervention being perceived as acceptable by patients and therapists, and identification of barriers and facilitators to remote delivery. Further details are shown in online supplemental table 1.

### Feasibility study participants

#### Patients

All eligible patients admitted to major trauma centres at Queen’s Medical Centre, Nottingham and The Royal London Hospital between October 2020 and January 2021, were invited to participate via post, telephone or in person (see figure 1). Eligibility criteria were patients aged 16–69 years, with an injury severity score >8, in work (paid or voluntary) or full-time education prior to injury, with no plans to retire within the next year, sufficient English language proficiency to contribute to the data collection or willing to use an approved interpreting service, resident in major trauma centre catchment area and able to give informed consent. Exclusion criteria were: participation in other vocational rehabilitation trials or those who had returned to work or education for at least 80% of preinjury hours. Those giving informed consent to participate completed a baseline questionnaire (via video, telephone or email) and all were invited to participate in a postintervention interview approximately 3 months after the start of the intervention.

#### Therapists

Therapists were identified through their managers and through the research team’s networks. 12 months after initial recruitment of therapists, an additional OT was recruited to replace an OT who left the study. This replacement OT was not part of the initial training workshop and competency assessment described above. Instead, they received bespoke remote training and an adapted remotely delivered competency assessment. All recruited therapists gave informed consent to participate in pretraining and post-training questionnaires and interviews.

### Procedure

#### Initial training and pretraining and post-training questionnaires

The initial training workshop was held in January 2020. Prior to the training, therapists were sent a case study to read and complete questions about, and relevant research papers. At the start of the workshop, they completed a pretraining questionnaire on work experience, current role and previous training. Post-training, they completed a questionnaire on usefulness and experiences of the training, attitudes towards evidence-based practice (Evidence-Based Practice Attitude Scale 15 item version),^49 50^ confidence in undertaking evidence-based practice (4 items from the Evidence Based Practice Confidence Scale)^51^ and bespoke questions on confidence in providing the ROWTATE programme (information on measures is provided in online supplemental table 2). Therapists were provided with the ROWTATE intervention manual, case report form completion instructions, examples of return-to-work plans and other useful resources (eg, local services and sources of support for patients). The training included team introductions, an overview of the ROWTATE research programme, normal trauma responses and post-traumatic stress disorder, familiarisation with the intervention manual, working through a case study using the intervention manual, practising worksite assessment and practising completion of case report forms. The workshop ended with a competency assessment. Training was delivered by the two mentors (experienced OT (JH) and CP (NS), CP expert in post-traumatic stress disorder (SR), and co-chief investigator (DK)). The training was facilitated by JK, KR and BK.

### Patient and public involvement

Patient and public involvement representatives with lived experience of traumatic injury took part in the workshop to provide the patient perspective on intervention delivery.

### Competency assessment

Competency was assessed at the end of the initial 2-day initial training workshop using three bespoke structured tasks.

Task 1: Reviewing patient baseline data and collecting additional injury, health and work information through face-to-face interaction with the ‘patient’. This task was assessed using a team objective structured clinical examination,^52^ with the team comprising OTs and CPs from a study site. The team objective structured clinical examination was assessed using an observation marking sheet developed for the study. Therapists were observed before and during the meeting with a trauma ‘patient’, who was played by an experienced team objective structured clinical examination actor. The ‘patient’ followed a vignette depicting return-to-work after a lower limb fracture and wore props (leg brace or wheelchair) to make the exercise more realistic. The vignette depicted injury and work details before and after the injury. The ‘patient’ was instructed to report issues with their physical and psychological health and not to report some details unless specifically asked. The ‘patient’ was given potential questions and prepared answers prior to the assessment. Prior to the assessment, therapists were given the ‘patient’ self-completed baseline questionnaire containing demographic details, injury details, hospital admission details, preinjury and postinjury employment/education details, quality of life, health status (pain, physical functioning, anxiety, depression, post-traumatic stress, mental functioning), recovery expectations, work ability, financial distress, return to work plans and health service use).

Task 2: Writing a management plan which was assessed by comparing management plans against an expert developed OT/CP management plan. Therapists were provided with site-specific trauma pathway maps outlining rehabilitation and other health services available to patients in that area^34^ required for tasks 2 and 3 and a blank case formulation sheet, currently in use in the National Health Service for task 2.

Task 3: Writing a letter to employers about the patient. This was assessed by comparing letters against a series of questions designed by expert OTs/CPs.

Tasks were scored by mentors OT (JH) /CP (NS) who were provided with an instruction sheet and the opportunity to discuss the process prior to the assessment. A second independent assessor (BK, JK and 2 OT experts in vocational rehabilitation (JP, RT)) scored tasks to increase the rigour of the assessment process.

### Literature review

A qualitative telerehabilitation literature review was conducted to explore barriers and facilitators affecting delivery of remote interventions (search terms are shown in online supplemental table 2). Medline, EMBASE and PsychINFO were searched in June 2020. Qualitative studies of any design were included if they reported actual rehabilitation interventions delivered online, via phone, computers or other technologies. The search focused on patients with any type of physical injury (including traumatic injuries and acquired brain injury) and interventions that were delivered by any healthcare practitioner. Studies comprising automated or computerised interventions (eg, rehabilitation apps, computer programmes or games) without any contact with rehabilitation providers were excluded because of lack of applicability to the ROWTATE intervention. Studies that did not report actual interventions were also excluded.

### Preadaptation training questionnaires

Therapists completed questionnaires before the adapted training. The questionnaire collected information on determinants of health and professional behaviour of therapists that may influence intervention delivery. The questionnaire was informed by the Theoretical Domains Framework^53^ and comprised 14 behavioural determinants domains capturing beliefs, behaviours, emotions, skills, etc (see online supplemental table 1 for details of the measures used).

### Preintervention interviews

Prior to attending the adapted training, therapists participated in a semistructured interview between July and August 2020. The interviews aimed to explore views and experiences of telerehabilitation to identify factors that might influence implementation. Interviews also covered views on remote training and how the initial face-to-face training could be adapted for remote delivery (see online supplemental table 3 for copies of topic guides)

### Adapting the training and intervention

We collaborated with Australian colleagues who successfully pioneered vocational telerehabilitation.^54 55^ Data from the literature review, questionnaires and interviews were used to adapt intervention components and the training package for remote delivery. This included pretraining preparation and a practice session using Microsoft Teams with OTs/CPs, adapting work history taking, assessing workability and work hardening and developing telerehabilitation-specific work hardening templates, initial assessment forms, ideas for conducting remote workplace assessments, remote delivery set-up and preparation and completing adapted intervention case report forms.

The same therapists who were trained to deliver the face-to-face intervention prepandemic attended additional training to deliver the adapted intervention. This training was delivered by video conferencing (Microsoft Teams) over two half-days in September 2020. Training included pretraining preparation and practice sessions using Microsoft Teams, preparing and setting up for remote delivery of the intervention, remote assessment of work function, remote conduct of work hardening and workplace assessments, completion of adapted case report forms and local application of learning. The training was delivered by a research fellow (JK), co-chief investigator (KR) and the OT mentor (JH).

Refresher training was provided for therapists in June 2021. This comprised a remotely delivered 2-hour session that included a reminder of the intervention processes, feedback of interim fidelity results, good practice examples and sharing further resources.

### Postadaptation training questionnaires

Therapists completed questionnaires containing the same questions as the preadapted training questionnaire within 1 month of the adapted training.

### Intervention delivery

Sample sizes of 40–50 participants in total have been recommended for feasibility studies.^56 57^ We considered that 40 participants were sufficient to address the (original) objectives of the feasibility study (described above). The original objectives of the feasibility study did not include estimation of parameters to inform a sample size calculation for the main trial.

During the pandemic, the feasibility study had to be adapted, and the adapted objectives are described above. The adapted feasibility study predominantly involved the collection of in-depth qualitative data and process data (eg, number of intervention sessions provided, length of sessions, duration of intervention provision), as well as some descriptive quantitative data (eg, characteristics of study participants). We considered that 10 patient participants were sufficient to enable us to address the objectives based on the concept of information power.^57^ The concept of information power suggested that sample size choice should depend on the content of the sample as well as size, and it can be reduced in qualitative studies if certain criteria are met, which was the case in our study. These include the aims of the study being narrow, the sample being very specific to the objectives, using established theory in the analysis (we used the theoretical domains framework and the theoretical framework of acceptability), using experienced interviewers and not proposing a cross-case qualitative analysis. The objectives of the adapted feasibility study did not include estimation of parameters to inform a sample size calculation for the main trial or to make any statistical comparisons. Similar interview numbers can be found in other feasibility studies.^54 58^

Furthermore, we considered that it would be possible to recruit and deliver the intervention to 10 participants during the pandemic, despite National Health Service hospitals pausing non-COVID-19 related research for some of the pandemic, redeployment of therapists to other roles and limitations on researcher access to patients. Participants completed baseline questionnaires (between November 2020 and January 2021) collecting information on demographic, injury and employment or education details, employer characteristics, sickness absence and pay, workability, health-related quality of life with 5 level responses version (EQ-5D-5L),^59^ depression and anxiety (Hospital Anxiety and Depression Scale),^60^ post-traumatic stress (Impact of Event Scale-Revised^61^) and a cognitive assessment (Montreal Cognitive Assessment score).^62^ Detailed information on the scales can be found in online supplemental table 1. Therapists delivered the intervention to participants for up to 12 months (between December 2020 and January 2022), dependent on participant need and completed case report forms and additional intervention notes detailing intervention delivery mode, frequency, duration and content. Mentors completed a mentoring record for each mentoring session covering clinical issues, implementation issues, therapist issues, trial-related issues including contamination and potential harms of the intervention. The mentoring record was based on research findings identifying factors affecting delivery of a vocational rehabilitation intervention for people with traumatic brain injury.^58^

Fidelity of intervention delivery was assessed using data from case report forms, additional therapist intervention notes and mentoring records to complete a fidelity checklist for each participant who received the intervention. The checklist was adapted from the one used in a trial of a vocational rehabilitation intervention in stroke patients,^63^ by incorporating OT process components from the ROWTATE intervention logic model and adding clinical psychology components. Components were defined as ‘core’ (those within the control of the therapist that should be delivered, eg, screening for fatigue) or ‘desirable’ (those outside the therapist’s control, e.g., educating the employer which should be delivered if feasible). The OT intervention had 41 components overall (32 core and 9 desirable components) and the CP intervention had 37 components overall (27 core and 10 desirable components). The checklist is divided into four intervention stages: Stage 1: early recovery, stage 2: graded return to work, stage 3: job retention and stage 4: discharge process. A postgraduate psychology researcher (RL) completed the checklist after receiving training by an experienced OT (JH) on conducting fidelity assessment. Adverse events were collected by participants and therapists returning adverse events reporting forms.

### Postintervention interviews

Approximately 3 months after the start of the intervention, semistructured interviews were conducted remotely via Microsoft Teams or phone with consenting therapists and patients. Interviews explored acceptability of the intervention and factors that might influence its delivery, including views on remote intervention delivery and on the usefulness of the training. Interview guides were informed by the Theoretical Domains Framework^53^ and the Theoretical Framework of Acceptability^64^ and the telerehabilitation literature review. Interviews were conducted between August 2020 and March 2021 by four postgraduate researchers (JK, RL, KB and PP) with experience and training in psychology, trauma and/or rehabilitation who were provided with training from an experienced qualitative researcher (ST).

### Analysis

#### Pretraining and post-training questionnaires

Data were analysed using means (SDs) and medians (IQRs) as appropriate. Evidence-Based Practice Attitude Scale scores were calculated as described by Aarons^49^ and are presented as means (SDs). Categorical data were described using frequencies and percentages. Missing Evidence-Based Practice Attitude Scale data was dealt with based on authors’ instructions for each scale (Aarons GA, Personal email communication, 2022).

#### Competency assessment

The scores for each task were calculated and analysed separately. Scores for each task could range from 0 to 64 for task 1, 0–9 for task 2 and 0–1 for task 3. Weighted scores (40% for task 1, 50% for task 2 and 10% for task 3) were combined to calculate a total score. Total scores were categorised into competent (≥50%) or needing additional support (≤49%). The percentage agreement between independent assessors was calculated via kappa statistics. Statistical analysis of agreement was not undertaken due to small numbers.

#### Literature review

Studies were synthesised narratively. Qualitative findings were mapped to the framework for conceptualising drivers of and barriers to health IT adoption^65^ and the Theoretical Domains Framework.^53^

#### Preadaptation training questionnaires

Theoretical Domains Framework survey data were analysed using means (SDs). Barriers were defined as a mean domain score of ≤3.5 and facilitators as a mean domain score of ≥5.^53^

#### Preintervention interviews

Interviews were audio recorded and transcribed, then analysed using framework analysis^66^ (comprising five steps: transcription, familiarisation, coding, developing the framework and applying the framework). The framework was informed by the Theoretical Domains Framework.^53^

#### Postadaptation training questionnaires

The same analysis was used for the postadapted training questionnaires as for the preadaptation training questionnaires (see above).

#### Intervention delivery and fidelity

The number, frequency, duration and content of sessions was described using means (SD) or medians (IQR) as appropriate for continuous data and percentages for categorical data. Adherence to the intervention was measured using:

- Attendance (the percentage of sessions offered vs number rearranged by (1) patient participant and (2) OT or CP).
- The percentage of sessions delivered remotely.

Fidelity was assessed by calculating the percentage of all core and desirable components delivered excluding non-deliverable components (eg, employer education could not be provided if the participant refuses to consent to employer contact). Fidelity percentages are presented for the four stages of the intervention and for the overall intervention.

#### Postintervention interviews

Interviews were audio recorded and transcribed, then analysed using framework analysis^66^ (comprising five steps: transcription, familiarisation, coding, developing the framework and applying the framework). The framework was informed by the Theoretical Domains Framework^53^ and the Theoretical Framework of Acceptability.^64^

#### Integration of methods

Integration of methods occurred during the data analysis and interpretation stages, which occurred concurrently. Our interpretations were borne from comparing and contrasting findings from each dataset for each objective. Finally, we used a narrative synthesis approach, whereby we wove together the key findings from the different approaches, based on the Theoretical Domains Framework^53^ and the Theoretical Framework of Acceptability.^64^ Discrepancies or ‘unexpected’ findings were discussed with the wider research team to make sense of the findings and why divergence occurred.

## Results

### Objective 1: evaluation of therapist training for the original face-to-face intervention

#### Training evaluation results

Six therapists (four OTs and two CPs) took part in the initial group training, and a seventh therapist joined the study later and received individual training. Characteristics of the therapists reported on the pretraining questionnaire are shown in table 2.

The posttraining questionnaire results are shown in table 3. Therapists found the training useful and that it provided them with new knowledge, respected their previous knowledge, used an appropriate mix of different activities, the trainers’ approach was helpful and joint training with OTs and CPs was useful. The lowest score was for having unanswered questions or uncertainties at the end of the training, indicating that most respondents did not have unanswered questions or uncertainties at the end of the training.

**Table 3:** Experiences of training, attitudes towards and confidence in evidence-based practice and confidence in delivering the ROWTATE intervention

Measure	Item or domain	Median (IQR) unless otherwise stated
Experiences of training	Usefulness of training	4.0 (4.0, 5.0)
	Training content was what expected	4.5 (4.0, 5.0)
	I learnt something new from the training	5.0 (5.0, 5.0)
	The training was provided in a way that respected what I already knew	4.5 (4.0, 5.0)
	Training OTs and psychologists together were useful	5.0 (5.0, 5.0)
	The training was too long	2.0 (2.0, 2.0)
	The trainers’ approach was helpful	5.0 (5.0, 5.0)
	The training wasn’t interactive enough	2.0 (2.0, 2.0)
	The mix of different activities in the training was about right	4.5 (4.0, 5.0)
	I still have unanswered questions or things I am unsure about at the end of the training	3.0 (4.0, 3.0)
Evidence-based practice attitude scale	Dimension 1—Intuitive appeal of EBP	Mean=2.2 (SD=0.8)
	Dimension 2—Likelihood of adopting EBP given requirements to do so	Mean=2.7 (SD=0.6)
	Dimension 3—Openness to new practice	Mean=2.9 (SD=1.1)
	Dimension 4—Perceived divergence between research-based/academically developed interventions and current practice (low score indicates little divergence)	Mean=0 (SD=0.6)
	Overall score (Dimension four is reversed when calculating the overall score)	Mean=2.9 (SD=0.9)
Evidence-based practice confidence	Determine if evidence from the research literature applies to your/patient’s situation?	75.0% (70.0%, 90.0%)
	Ask your patient about his/her needs, values and treatment preferences?	100.0% (90.0%, 100.0%)
	Decide on an appropriate course of action based on integrating the research/evidence, clinical judgement and patient preferences?	90.0% (80.0%, 90.0%)
	Continually evaluate the effect of your course of action on your patient’s outcomes?	90.0% (90.0%,100.0%)
Confidence in delivering the ROWTATE intervention	Provide the ROWTATE programme to patients with traumatic injuries	80.0% (70.0%, 80.0%)
	Work with other colleagues (eg, psychologist/OT) who are providing the ROWTATE programme for patients	100.0% (90.0%, 100.0%)
	Liaise with employers about the patient returning to work	80.0% (80.0%, 90.0%)
	Liaise with other people involved in the patients care, for example, physiotherapists, solicitors etc	80.5% (80.0%, 90.0%)
	Refer or signpost the patient to other agencies as needed for example, Improving Services to Psychological Therapies (IAPT), self-help groups, other sources of support	80.0% (80.0%, 90.0%)
	Seek advice and support from your ROWTATE mentor	100.0% (90.0%, 100.0%)
	Identify issues that may lead to contamination in the study	80.5% (80.0%, 90.0%)
	Complete the paperwork required for the project	90.0% (80.0%,90.0%)

EBP, evidence-based practice; OT, occupational therapist; ROWTATE, Return to Work After Trauma.

The mean total Evidence-Based Practice Attitude Scale score was 2.87 (SD=0.93; possible score range 0–4) indicating positive attitudes towards evidence-based practice. Median Evidence Based Practice Confidence Scale scores ranged from 75% (IQR 70%–90%) to 100% (IQR 90%–100%) across the four items across the four questions indicating high levels of confidence to deliver evidence-based practice. Median confidence scores in delivering aspects of the ROWTATE intervention ranged from 80% (IQR 70%–80%) to 100% (IQR 90%–100%) across the eight questions indicating high levels of confidence in delivering the intervention.

#### Competency assessment results

Competency assessments were conducted for the six therapists who took part in the initial training. 50% of therapists were judged to be competent for task 1 (reviewing baseline data and collecting additional information assessed by team objective structured clinical examination observation), 100% for task 2 (assessed by management plan) and 83% for task 3 (assessed by letter to employer). Therapists assessed as needing additional support were provided with this through mentoring and additional training (eg, how to write reports about return to work plans) to address their competency needs. Assessors agreed on competency ratings (competent vs needs support) for 50% of the team objective structured clinical examination observations (task 1), 100% of the management plans (task 2) and 67% of the letters (task 3).

### Objective 2: adapt the ROWTATE OT and clinical psychology training to make it suitable for remote delivery

#### Literature review

Eight papers were included in the review. These included telerehabilitation interventions provided to patients with a range of injuries including brain injury (traumatic or acquired),^31 56 67 68^ spinal cord injury,^32^ orthopaedic trauma,^57^ multiple trauma^69^ and multiple conditions (burns, stroke and spinal cord injury).^33^ Telerehabilitation was provided through many different platforms and devices including video conferencing,^32 56 57 68^ telephone calls,^33 56 67 69^ automatic reminders^31^ and educational websites.^56^

Key barriers identified in the review included: technological issues (including poor access and quality of technology,^31 32 68^ infrastructure and organisation issues^68 69^ which caused delays to intervention delivery,^31 56^ and inhibited technology use.^56^ These issues were exacerbated by language barriers and older age.^57^ Lack of knowledge and comfort with technology^31^ and capacity for therapists or administrators to organise the rehabilitation delivery was a further barrier.^31 68^ Patient physical, psychological (eg, anxiety) and cognitive impairments or ability were also identified as factors which could make using telerehabilitation technology more difficult.^31 32 56 68^ Some intervention elements were seen by therapists as incompatible with telerehabilitation including physical examinations (eg, mobility training, strength),^31 57 68^ psychosocial components (eg, post-traumatic stress disorder) and interactions in group setting interventions.^69^ The need to redesign forms and procedures (eg, worker certificates)^57^ and develop new procedures around data storage and transfer^57^ was also identified as barriers by therapists. Finally, lack of staffing as a consequence of longer preparation time required (compared with face-to-face appointments) or need for administrative staff was a barrier.^32 57^

Key facilitators included training of patients,^33^ therapists and administrative staff in using telerehabilitation^56^ as well as providing support to use the technology.^33 57 68^ Other facilitators for staff included having a champion for telerehabilitation,^68^ organisational leader support,^32^ resources,^32 57^ appropriate space to conduct telerehabilitation appointments, systems in place that enable organising remote interventions,^68^ and using a combined approach whereby some of the intervention happened face-to-face.^31 32 56^ Technology that was simple and took account of patients’ abilities,^31 56^ shorter and more frequent meetings (to prevent fatigue),^31 67^ screen sharing enabling form completion and discussion of intervention plans,^31^ and use of computer imaging^57^ were also identified as facilitators. Positive experiences of use of telerehabilitation or knowledge exchange,^31^ advance sharing of patient information when multiple sites are involved^33 68^ and good coordination^57^ also acted as facilitators for staff.

#### Preadaptation training questionnaire results (full details published elsewhere)

All therapists (four OTs and two CPs initially trained, plus one additional OT who joined the study later) completed the preadaptation training questionnaire. Only one Theoretical Domains Framework domain was identified as a barrier, which was therapists’ intentions to use remote vocational rehabilitation (mean 3.4 (SD 0.23)). Two domains were identified as facilitators, which were social influences (mean 5.0 (SD 0.55)) and knowledge (mean 5.03 (SD 0.34)).^42^

#### Preintervention interviews with therapists (full details published elsewhere)

All therapists (four original OTs and two CPs, and the additional OT joining 12 months later) participated in preintervention interviews. These identified several barriers to remote delivery which included: access to technologies, reduced ability to assess an individual’s environment remotely, challenges with conducting functional, cognitive or workplace assessments remotely, patients’ lack of ability or confidence in using technology, change in professional role and identity given the new form of delivery, and difficulty building therapeutic rapport online.^42^

#### Adapting the training

The training was adapted to increase therapist confidence in delivering the remote intervention by providing resources to support online sessions, examples and live training on using the resources via Microsoft Teams (eg, Whiteboard, Padlet, share screen function), sharing concerns or experiences of remote delivery and identifying ways to overcome barriers. This encouraged peer support and rapport building between therapists. The training was also adapted to include a session on the effectiveness, acceptability and potential benefits of telerehabilitation, informed by the findings of the literature review. The therapist intervention manual was adapted for remote delivery of the intervention and included resources to support remote delivery, tips for building rapport online and examples of remote assessments. Adaptations to the training were informed by discussions with patient and public involvement, the literature review and therapist interviews.

#### Postadaptation training questionnaire results (full details published elsewhere)

Four therapists (three OTs, one CP) completed the postadaptation training questionnaire. Following the adapted training, 13 of the 14 domains were identified as facilitators and no barriers were identified.^42^

#### Postintervention interviews with therapists

Postintervention interviews were conducted with six therapists (four OTs and two CPs as one OT withdrew prior to the end of the study). Therapists found the training useful and all felt it sufficiently prepared them to deliver the ROWTATE intervention remotely:

I think it [training] was really useful in orienting us to the platforms, the technology that we could use, some of the issues that can come whilst using the technology, and I guess the purpose and the focus of the intervention, particularly as that had kind of shifted if we think about the remote variant of ROWTATE. That was a real shift from, I guess, how we were expecting to be delivering the study and it was useful to just emphasise that we're really trying to learn about whether this can work remotely and, actually, there might be some benefits from doing it this way.’ (Clinical psychologist)

Yeah, I think it [training] was very thorough. As things have gone along and having mentoring with [ROWTATE mentor], there are things down the line that you still need to ask questions about because you don't take everything on board…So I think you did a relatively good amount of getting us ready and prepared for it, but it’s like anything, until you actually start doing it, that’s when you start asking questions.’ (Occupational therapist)

Training OTs and CPs together fostered good working relationships between OTs and CPs and most therapists preferred face-to-face training because this enabled development of the therapeutic OT/CP team:

There was something, wasn’t there, about the way that we all came together for that first training [face-to-face) and you kind of had that space to just chat between sessions and form as a bit of a group in that way. So, I wonder if your first training was remote, if it'd be that much harder to bring everybody together. (Clinical psychologist)

All therapists felt that a blended approach to training incorporating face-to-face and remote training would be preferable for the definitive trial.

I’d like a bit of both [remote and face-to-face training], but erring more on the remoteness, because of the efficiency of it. (Occupational therapist)

### Objective 3: adapt the ROWTATE intervention to make it suitable for remote delivery via telerehabilitation and telepsychology

#### Adapting the intervention

The intervention was adapted to be delivered predominantly by remote delivery (videocall or phone). This included the development of a detailed remote initial patient assessment form, work hardening templates and ideas for conducting remote workplace assessments. This included a specific workplace assessment guide. A description of the adapted intervention is given in online supplemental table 5. Findings relating to factors affecting delivery and acceptability of the remote intervention to patients and therapists have been published elsewhere.^42^

### Objective 4: deliver the adapted ROWTATE intervention and assess feasibility and fidelity of remote delivery via telerehabilitation and telepsychology

#### Characteristics of patients receiving the intervention

**Figure 2:**
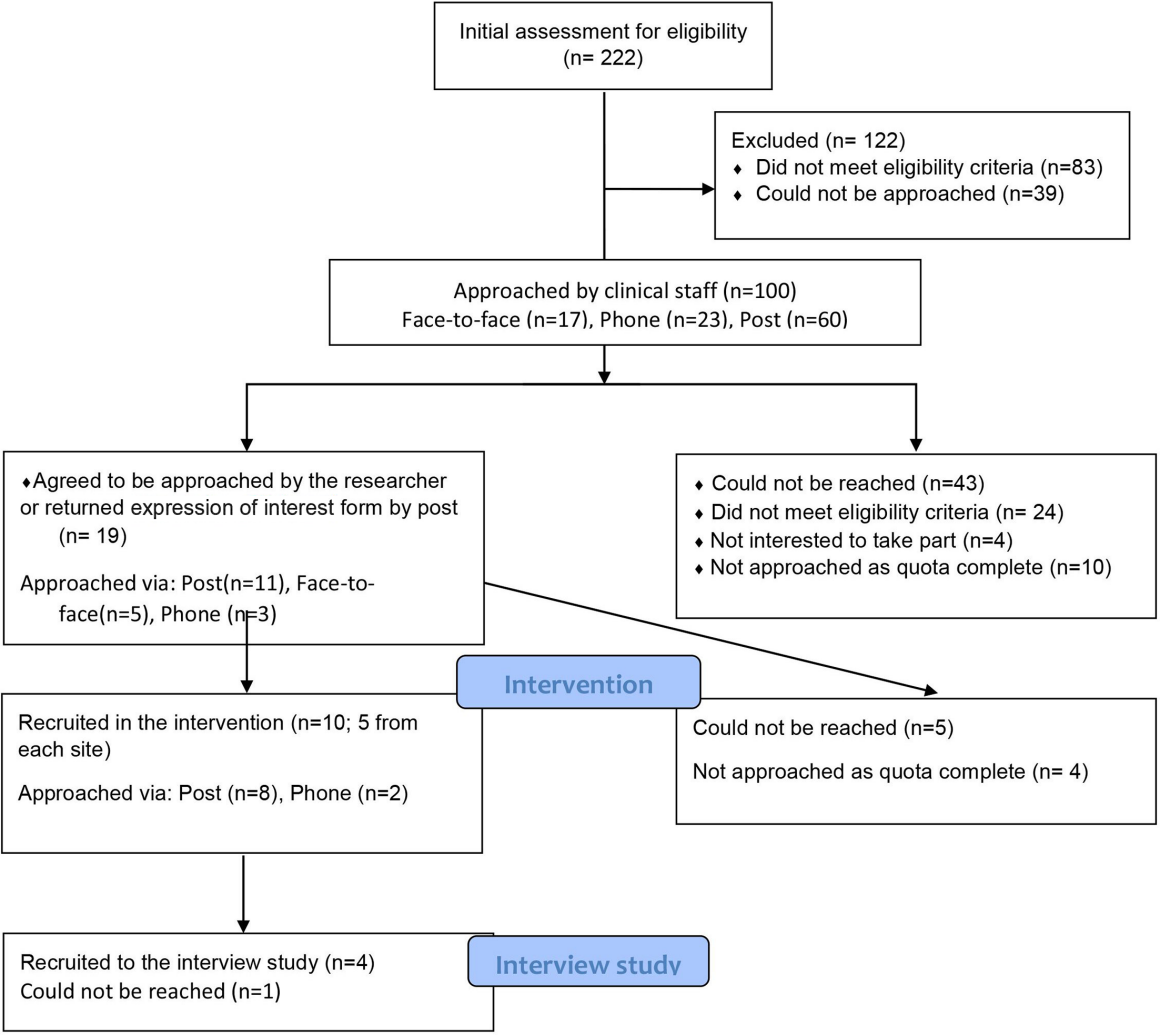
Recruitment flow diagram. This diagram outlines the recruitment processes for both the intervention and interview study aims. Of the 222 traumatic injured patients assessed for eligibility, 122 were excluded due to not meeting the eligibility criteria (n=83), or for being unreachable (n=39). Overall, 100 patients were approached by clinical staff face-to-face (n=17), phone (n=23) or post (n=60). Of these, 19 patients agreed to be approached by the researcher or returned the expression of interest form, leading to 10 participants being recruited in the intervention (5 from each site). Finally, of the participants who took part in the intervention, four took part in the interview study.

100 patients were approached and the required number of patient participants were recruited (n=10, 10% of those approached) to receive the intervention (5 from each study centre) (figure 2) over 2 months (1 October 2020–5 May 2020). Characteristics of participants are shown in table 4. The median time from injury to recruitment was 58 days (IQR 37.3, 62.8). The median age of participants was 41.0 years (IQR 31.0, 54.8), 60% were male, participants had a median of 18 years of full-time education (IQR 12.8, 20.5) and 40% were from an ethnic minority group. 40% were in paid employment only, 20% were self-employed only, 10% were in full-time education only and 30% had multiple jobs/education. 70% were injured in road traffic crashes, the median injury severity score was 11 (IQR=9, 17) and the median length of hospital stay was 6.5 days (IQR 5.0, 18.0). The median EQ5D-5L utility score was 0.46 (IQR 0.17, 0.66), Hospital Anxiety and Depression Scale–Anxiety was 9.0 (IQR 5.2, 12.3), Hospital Anxiety and Depression Scale–Depression 6.0 (IQR 4.0, 10.0), Impact of Event Scale-Revised 26.5 (IQR 11.5, 38.0) and Montreal Cognitive Assessment score was 27.0 (IQR 25.0, 28.0). One patient (10%) reached the threshold criteria for depression (>10), five (50%) for anxiety (>10) and four (40%) for symptoms of post-traumatic distress (>32). Eight participants (80%) returned to work during the intervention period. This is higher than the return to work rate in similar UK studies.^16 70^

**Table 4:** Baseline characteristics of participants receiving the intervention

Characteristic	Frequency (%) or median (IQR)
Age	41.0 (31.0, 54.8)
Gender	
Male	6 (60%)
Female	4 (40%)
Ethnic group	
White	6 (60%)
Asian	3 (30%)
Black	1 (10%)
Marital status	
Married/civil partnership	6 (60%)
Single	3 (10%)
Divorced/widowed/separated	1 (10%)
Years of full time education	18.0 (12.8, 20.5)
Employment/education	
Employed	4 (40%)
Self employed	3 (30%)
Full-time education	1 (10%)
Employed and self-employed	1 (10%)
Employed and full-time education	1 (10%)
Occupation	
Skilled agricultural, forestry and fishery workers	1 (10%)
Services and sales workers	5 (50%)
Craft and related trades workers	1 (10%)
Managers	1 (10%)
Technicians and associate professionals	1 (10%)
Professionals	1 (10%)
Days from injury to recruitment	57.9 (37.3, 62.8)
Length of hospital admission (days)	6.5 (5.0, 18.0)
Injury Severity Score	11.0 (9.0, 16.5)
Body region injured*	
Head	6 (60%)
Thorax	4 (40%)
Abdomen	2 (20%)
Spine	2 (20%)
Upper limb	2 (20%)
Lower limb	3 (30%)
Mechanism of injury	
Road traffic crash	7 (70%)
Struck by person/object	2 (20%)
Fall	1 (10%)
Hospital Anxiety and Depression Scale–Anxiety score	9.0 (5.2,12.3)
Hospital Anxiety and Depression Scale–Depression score	6.0 (4.0, 10.0)
Impact of Event Scale-Revised score	26.5 (11.5, 38.0)
Health-related quality of life (EQ5D) utility index	0.46 (0.17, 0.66)
Montreal Cognitive Assessment score	27.0 (25.0, 28.0)

* Some participants had multiple injuries.

#### Intervention delivery

##### Commencing the intervention

All participants were seen by the OT within 2 weeks of recruitment to the study (median 8.5 days, IQR 6.5, 11.5). Nine (90%) participants were seen by the OT within 12 weeks of injury (median 9.3 weeks, IQR 7.5, 10.2). Seven (70%) participants were assessed by the CP with a median of 30.0 days (IQR 25.0, 55.0) from recruitment and a median of 13.0 weeks, IQR (10.6, 16.6) from injury.

##### Dose (duration and frequency)

The OTs’ intervention within the study was delivered between November 2020 and January 2022. Participants received the OT intervention for a median of 34.6 weeks (range, 21–51 weeks). CPs delivered the intervention from 16 December 2020 to 15 December 2021. Participants received the CP intervention for a median of 7.9 weeks (IQR 0.1, 34.6).

OTs delivered a median of 14.25 hours (IQR 10.9, 17.9) of intervention per participant during a median of 13 sessions (IQR 9.5, 14) per participant. Session duration was between 15 and 140 min, a median of 60 min per session (IQR 50, 75). In six out of seven participants, CPs delivered a median of 3.5 hours (IQR 1.3, 6.5) of intervention per participant during a median of 3 (IQR 1, 8.5) sessions per participant. Session duration was between 10 and 127 min; a median of 45.5 min per session (IQR 35, 61.3). There were missing intervention session data for one participant.

A total of 136 OT sessions were delivered to the 10 participants across the 12-month period. Most sessions were delivered early in the intervention period, with the highest frequency of sessions per participant in month 2 (median 2.5 sessions; IQR 2, 3.8). Seven participants remained on the OTs’ caseload for more than 6 months. Only one participant remained on the OT caseload for 12 months.

Seven participants (70%) were referred by the OT to the CP and received a total of 37 CP sessions across the 12-month period. Most sessions were delivered early in the intervention, with the highest frequency of sessions per participant in month 2 (median 1 session, range 1–2 sessions). Three participants received only one CP session. Three participants remained on the CP caseload for more than 6 months. Only one participant remained on the CPs’ caseload for 12 months.

##### Adherence (attendance and use of remote delivery)

The median percentage of OT sessions attended was 97.2% (IQR 83.3, 100). Patients attended all planned CP sessions. In total, 97.8% (n=133) of OT sessions and 100% (n=37) of CP sessions were delivered remotely. Three face-to-face sessions were conducted because one participant reported falling at home and the OT wanted to investigate further; one participant required technical assistance to set up video conferencing and one participant specifically requested an in-person meeting.

Of the 133 remote sessions delivered by OTs, the remote systems used were: phone for 39 (29.8%) sessions, videocall via DrDoctor for 38 (29.0%) sessions, videocall via Microsoft Teams for 32 (24.4%) sessions and videocall via Zoom for 22 (16.8%) sessions (missing data on 2 sessions). Of the 37 CP sessions, 16 (48.6% were delivered by phone, 5 (14.3%) by DrDoctor, 10 (28.6%) by Microsoft Teams and 4 (11.4%) by Zoom (missing data on 2 sessions).

##### Withdrawal from the intervention

None of the participants withdrew from the intervention.

##### Intervention content

The median number of minutes spent delivering each intervention component is shown in table 5. For OTs, most time was spent dealing with current issues (eg., medical, social, financial, family issues), work preparation, return to work without direct contact with employer or education provider, fatigue/pain management and dealing with medical issues (eg, appointment issues, healthcare problems). The least amount of time was spent setting/reviewing homework, providing family/carer support, providing assessment/intervention on activities of daily living or cognition. OTs did not attend medical appointments with any participants or undertake job redirection activities. For CPs, most time was spent on interrogative thinking and return to work without direct contact with employer/education provider. The least amount of time was spent on setting/reviewing homework, providing family/carer support and job redirection. No participants received assessment/interventions for activities of daily living or cognition, and CPs did not attend medical appointments with patients or monitor job retention.

**Table 5:** Minutes of occupational therapy and clinical psychology intervention components delivered

Components	Median minutes of intervention component delivery per participant (IQR)		Median minutes of intervention component delivery per participant (IQR)	
	OT (n=10 patient participants)	Number of patients receiving each component	CP* (n=6 patient participants)	Number of patients receiving each component
Early recovery assessment	20.0 (15.0, 30.0)	10 (100.0%)	20 (17.5, 30.0)	3 (50.0%)
Work-related assessment	23.0 (27.5, 35.0)	7 (70.0%)	Not applicable	
Current issues	100.0 (42.5, 162.5)	10 (100.0%)	40.0 (23.0, 90.0)	5 (83.3%)
Plan/review/monitor goals	20 (2.5, 62.5)	7 (70.0%)	16.0 (10.0, 26.0)	5 (83.3%)
Set/review homework	5.0 (0, 7.5)	7 (70.0%)	10.0 (10.0, 20.0)	5 (83.3%)
Family/carer support	10 (2.5, 10.0)	7 (70.0%)	10.0 (10.0, 10.0)	1 (16.6%)
Physical assessment/intervention for physical dysfunction	45 (12.5, 75.0)	8 (80.0%)	Not applicable	
Activities of daily living	10 (2.5, 25.0)	6 (60.0%)	Not delivered	0 (0.0%)
Mobility	35 (2.5, 87.5)	7 (70.0%)	Not applicable	
Cognitive assessment/intervention	5.0 (0, 27.5)	5 (50.0%)	Not delivered	0 (0.0%)
Psychological screening	40 (30.0, 50.0)	10 (100.0%)	Not applicable	
Emotional/psychological issues	55 (47.5, 100.0)	9 (90.0%)	31.0 (12.5, 44.25)	6 (100.0%)
Fatigue/pain management	65 (57.5, 82.5)	10 (100.0%)	13.5 (10.25, 25.75)	4 (66.7%)
Work preparation	105 (25.0, 150.0)	10 (100.0%)	Not applicable	
Dealing with medical issues	60 (12.5, 62.5)	9 (90.0%)	15.0 (5.0, 25.5)	4 (66.7%)
Attending appointments with participant	Not delivered	0 (0.0%)	Not delivered	0 (0.0%)
Return-to-work without direct employer/education provider contact	75 (42.5, 115.0)	8 (80.0%)	60.5 (34.0, 80.75)	4 (66.7%)
Return-to-work with direct employer/education provider contact	45 (0, 110.0)	5 (50.0%)	31.0 (21.5, 40.5)	2 (33.3%)
Monitoring job retention	50 (35.0, 60.0)	8 (80.0%)	Not delivered	0 (0.0%)
Job redirection	0 (0,0)	1 (10.0%)	5.0 (7.5, 12.5)	2 (33.3%)
Interrogative thinking	Not applicable		92.5 (71.0, 128.75)	4 (66.7%)

* One patient had one session with the CP for which the intervention case report form was not completed.

CP, clinical psychologist; OT, occupational therapist.

### Mentoring

27 OT and 19 CP mentoring sessions were conducted with a total of six therapists (one therapist left the study before the seventh therapist joined the study). The total time to deliver OT mentoring was 22.0 hours. Sessions lasted for a median of 57.5 min (IQR 48.8, 60.0). The total time to deliver CP mentoring was 13.1 hours. Sessions lasted for a median of 45.0 min (IQR 30.0, 45.0).

### Fidelity

Fidelity of intervention delivery for each individual stage of the intervention and across all four intervention stages for OTs and CPs is shown in table 6. Across all four stages, a high percentage of core and desirable intervention components were delivered as planned (84.5% for OTs and 92.9% for CPs). The intervention stage with the lowest fidelity was the discharge process stage (57.1% for OTs and 75.0% for CPs).

**Table 6:** Fidelity of intervention delivery by OTs and CPs by individual intervention stage and across all four intervention stages

	Intervention stage	Median percentage of core and desirable components delivered (IQR)
OT (n=10 patient participants)	1: Early recovery	92.5 (87.0, 100)
	2: Graded return to work	83.3 (75.7, 96.9)
	3: Job retention	87.5 (75.0, 100)
	4: Discharge process	57.1 (50.0, 76.7)
	Overall fidelity	84.5 (79.9, 88.0)
CP (n=6 patient participants*)	1: Early recovery	77.8 (73.2, 95.5)
	2: Graded return to work	100 (100,100)
	3: Job retention	100 (100,100)
	4: Discharge process	75.0 (66.7, 79.2)
	Overall fidelity	92.9 (83.5, 97.9)

* One patient had one session with the CP for which the intervention case report form was not completed.

CP, clinical psychologist; OT, occupational therapist.

Of the 32 core components of the OT intervention, there was evidence that 17 were delivered to all participants, seven were delivered to ≥75% of participants, four were delivered to 50%–74% of participants and three were delivered to fewer than 50% of participants. The least commonly delivered core components were providing emotional support to participants’ families (no evidence of delivery to any participant), discussing disengagement from the intervention with the participants’ employers (evidence of delivery to 25% of participants) and discussing how to re-access the intervention or providing information about further support with the participants’ employers (no evidence of delivery for any participant).

Of the 27 core components of the CP intervention, there was evidence that 16 were delivered to all participants, three were delivered to ≥75% of participants, two were delivered to 50%–74% of participants, two were delivered to fewer than 50% of participants and four were not delivered because the OT was leading on those components. The least commonly provided core components were recording participants’ goals 6 months postrecruitment, and providing a discharge letter to participants’ general practitioner/other relevant healthcare professionals (no evidence of delivery for either of these components for any participant). The four components which were not delivered because the OT was leading on these were negotiating phased return to work, addressing return to work issues with all stakeholders, discussing disengagement from the intervention with the participant’s employer, discussing how to re-access the intervention or providing information about further support with the participant’s employer.

### Adverse events

No adverse events were reported during the study. A complementary robust methodology was used to collect this data, which was developed in collaboration with patient and public involvement and project steering committee. The data collection method included patient self-report (where printed copies and envelopes were provided to patients at recruitment point), possibility to email or phone the researchers, as well as therapists and mentors reporting after the start of the intervention. Finally, interviews with patients and therapists provided a further opportunity to discuss any suspected adverse events.

### Factors influencing intervention delivery

Mentoring records showed that early in the study, there were common issues across both sites that affected the ease of remote delivery of the intervention. Factors hindering the delivery were in part due to COVID-19 restrictions and the need for new ways of working in the National Health Service. These included uncertainty about which video conference platform to use, local technical advice to support its use, therapist confidence to use it and some difficulty supporting participants to do the same. National Health Service Trusts rapidly changed policies about which platforms were permissible, requiring therapists to adapt to new platforms and reducing therapist confidence in remote delivery. Mentors supported therapists to deliver the intervention remotely by locating National Health Service Trust policies, providing telerehabilitation advice and making resources accessible on the ROWTATE website.

Some therapists were under considerable additional pressure in their pre-existing National Health Service jobs and found it challenging to remember the ROWTATE intervention process. Mentors spent time in mentoring sessions going through the intervention manual, demonstrating how to complete case report forms and where to locate online resources.

Discussing individual participants enabled mentors to identify difficulties in delivering and remind therapists of core intervention components, for example, OTs completing psychological screening, recording goals and reminders to rescreen participants, revisit goals and end the intervention on time. Mentors and therapists problem-solved issues, and mentors reminded therapists of example documents available on the ROWTATE website and provided feedback on written communications to employers to build therapist confidence (see table 7 for a summary).

**Table 7:** Factors facilitating or hindering delivery of core components of the intervention from the mentoring records

Barrier	Facilitator
Early in the study, there were common issues across both sites that affected the ease of remote delivery of the intervention: Covid restrictions New ways of working in the National Health Service that were not yet settled: Uncertainty about permissible video conference platforms Lack of local technical advice to support its use Low therapist confidence to use it Some difficulty supporting participants to access platforms. Rapidly changing policy about platforms exaggerated low confidence.	Mentors supported the therapists to deliver the intervention remotely by: Locating Trust policies Providing telerehabilitation advice Adding resources accessible on the ROWTATE website.
Additional pressure in therapists’ own jobs so found it challenging to remember to the ROWTATE intervention process.	Mentors spent time in mentoring sessions to: Go through the intervention manual Demonstrating how to complete case report forms, Where to locate online resources.
Individual caseload challenges such as participants not wanting: Support with return-to-work planning Therapist to write to employer, general practitioner, etc	Mentors and peers could problem-solve issues and provide advice. Therapists were reminded of the core components relative to each participant such as: OTs completing the psychological screening Recording goals Asking therapists to set calendar reminders to ensure they: Rescreened participants Revisited goals Ended the intervention on time. For written communications, mentors reminded therapists of: The examples available on the ROWTATE website Emailed them to the therapist Provided additional support such as feedback on written communication to an employer if this was new to the OT or CP with the aim of building the therapist’s confidence.

CP, clinical psychologist; OT, occupational therapist; ROWTATE, Return to Work After Trauma.

Other factors influencing intervention delivery were identified from postintervention interviews and have been reported elsewhere.^42^ In brief, the initial and adapted training and the experience of delivering the intervention addressed some therapist barriers to intervention delivery (eg, increased confidence in delivering the intervention remotely, ability to build good rapport with participants, acceptance of remote intervention delivery as part of professional role), but some barriers persisted. These included a range of IT issues for patients and therapists, the need for flexibility to enable face-to-face delivery where necessary, the need for strategies to enhance privacy and minimise disruptions during remote contacts between therapists and participants and the need for strategies to increase employer engagement.

### Progression criteria

Achievement of progression criteria is shown in online supplemental table 4. All criteria were achieved, except for assessing acceptability of, and barriers and facilitators to intervention delivery from the employers’ perspective. Strategies for addressing this are outlined in the discussion.

## Discussion

### Main findings

This feasibility study has shown that therapists found the training to deliver the intervention useful and at the end of training they had positive attitudes to and high levels of confidence in delivering evidence-based practice. They also had high levels of confidence in delivering the ROWTATE intervention. Half the therapists were judged to need some additional support in the competency assessment and were provided with this via mentoring or additional training. We identified a range of barriers and facilitators to remote delivery and used this information to adapt the intervention and training for it. Barriers identified by therapists before the adapted training became facilitators or ceased to be barriers after training. Nearly all intervention sessions were delivered remotely with very high attendance rates. The intervention was delivered with high levels of fidelity to core and desirable intervention components. No adverse events were reported during the study. Most criteria for progression to the definitive trial were met.

### Comparisons with evidence

An evaluation of a vocational rehabilitation training package for an intervention for patients with traumatic brain injury reported findings similar to ours in terms of usefulness of the training and OTs’ confidence to deliver the intervention post-training.^46^ We have not been able to find published studies reporting Evidence-Based Practice Attitude Scale or Evidence Based Practice Confidence Scale scores among OTs or CPs following vocational rehabilitation training.

The barriers and facilitators to remote delivery of the intervention identified in our study are similar to those identified in previous studies.^31 32 56 57 68^ This included issues around environmental context and resources, therapist knowledge and the key role played by mentors and peers supporting previous research.^49^ Our finding of high levels of fidelity of OT intervention delivery is consistent with those from a previous study assessing fidelity of a vocational rehabilitation intervention for people with traumatic brain injury.^58 63^

### Strengths and weaknesses

Strengths of the ROWTATE training included incorporation of patient perspectives on intervention delivery, training OTs and CPs from the same sites together enabling formation of good working relationships, consideration of local implementation issues and barriers and facilitators to intervention delivery, and providing pretraining materials to reduce time required for training.

We successfully recruited the 10 planned participants during the pandemic despite most patients being approached by telephone and/or post due to the pandemic restrictions and undertaking remote recruitment visits. This provided valuable experience for recruitment in the definitive trial if face-to-face recruitment visits are not possible. We recruited a diverse group of patients with a range of different injuries, injury severities, preinjury employment/education and occupations. While this number was changed following the adaptations of the study objectives and intervention delivery due to the pandemic, the revised methodology^69^,^71^ allowed us to answer the research questions.

Use of multiple data sources (questionnaires, interviews, case report forms, mentoring records) allowed qualitative data to enhance understanding of quantitative data. Use of data collection methods (eg, postintervention interviews) to address more than one objective minimised participant burden.

In terms of the training, sessions delivered didactically were less interactive and a prerecorded session could have been used instead. There was considerable disagreement between competency assessors, particularly in relation to the team objective structured clinical examination. At the core of these disagreements were the different competencies observed by the OTs and CPs which were informed by their professional background. The small number of therapists trained precluded statistical assessment of the level of assessor agreement. While assessors were provided with instructions and opportunity to discuss the assessment process, this may have been insufficient to ensure consistency of assessment. We measured attitudes towards and confidence in evidence-based practice only at the end of the training because the number of therapists was too small to allow statistical comparison of pretraining and post-training scores.

Although we recruited a diverse range of patients, our findings are limited to 10 patients. As the original aims of the feasibility study had to be changed due to the pandemic, we were unable to assess the effectiveness of recruitment and retention strategies, and a larger number of participants may have allowed more detailed understanding of intervention delivery and fidelity. Prior to the pandemic, our plan had been to recruit 40 participants to enable assessment of recruitment strategies, intervention delivery and collection of outcome data at 3 months follow-up. As a result of having to adapt the intervention for remote delivery, train therapists to provide the remote intervention and recruit participants remotely during the pandemic, the priority for the feasibility study changed to focussing on intervention adaptation and delivery. We considered a smaller sample size would be feasible to recruit under pandemic conditions and allow sufficient assessment of intervention delivery to inform decisions about progression to the definitive trial. Furthermore, the definitive trial design incorporates an internal pilot to assess recruitment and follow-up, so removing these components from the feasibility study during the pandemic seemed sensible.

The fidelity of the intervention was measured using data from therapist-completed case report forms and intervention notes. This therefore relied on accurate recording of the intervention. It is possible that therapists under-reported or over-reported intervention delivery. However, also obtaining data from intervention notes and mentoring records helped to corroborate data from the case report forms.

Employer engagement with the intervention and the research was more limited than expected. Some participants did not give consent for their employer to be contacted by the OT or by researchers, and others were self-employed and did not have an employer. The OT had direct contact with the employer for five participants and we were unable to interview any employers about their experiences of the intervention. Although 80% of participants returned to work despite fairly low levels of employer engagement, previous research suggests employer engagement is likely to be important to achieving a sustainable return to work,^24 71 72^ hence the need to develop strategies to increase engagement in the definitive trial.

### Implications for the definitive trial

#### Changes to therapist training

The Evidence-Based Practice Attitude Scale and Evidence Based Practice Confidence Scale scales will be measured pretraining and post-training, enabling the impact of training on these scales to be assessed. More use will be made of pretraining materials to reduce the didactic content of the training. A new online workshop will be provided to address questions about pretraining materials, study design, research contamination, telerehabilitation, OT/CP role and remote delivery and introduce therapists to the intervention case report forms. The training workshop will be conducted face-to-face and will focus on building rapport within and across site OT/CP teams, applying pretraining information to case study material, checking understanding of and practising key intervention processes and case report form completion. Patient and public involvement representatives contributed to all training workshops. Enhanced training for competency assessors, including practice scoring of tasks and comparison and discussion of scores between assessors, will be provided.

#### Changes to the definitive trial

Postal and telephone recruitment were the predominant recruitment methods used in the feasibility study due to pandemic restrictions. While this achieved the required small sample size, only 10% of those approached were recruited and recruitment took 2 months. Recruitment for the definitive trial will therefore predominantly be undertaken face-to-face, with phone and postal recruitment where this is not possible. This should increase the recruitment rate and reduce delays to commencing the intervention. It is clear that face-to-face contact between therapists and clients is sometimes necessary, hence the intervention will be delivered predominantly remotely with face-to-face contact as appropriate to patient need. This will help to address difficulties with remote assessments (eg., cognitive assessments or workplace assessments) and some barriers to use of technology. Agreement will be sought across participating hospital Trusts for therapists who deliver the intervention across sites to use the same software platform. Strategies to ensure privacy and minimise disruptions during therapist-participant contacts will be discussed in training and mentoring, as will strategies for therapists to increase employer engagement. We will pilot the use of observations of therapist-participant contacts to enable assessment of the accuracy of recording of intervention delivery. We will also work with patient and public involvement representatives who have experience as employers and employer representatives on our research programme steering committee to adapt employer-facing study documentation and develop events to increase employer engagement with the research aspect of the trial.

To ensure that core components of the intervention are delivered in the definitive trial, mentors will focus mentoring sessions on the intervention components found to have lower fidelity in the feasibility study (emotionally supporting participants’ families, discussing disengagement from, and how to re-access the intervention with employers, recording participants’ goals 6 months postrecruitment and providing discharge letters to general practitioners/other relevant healthcare professionals). OTs and CPs will also complete online case report forms that can be viewed in real time by mentors to facilitate individual supportive conversations by problem-solving any barriers to fidelity.

Best practice examples, case studies and intervention resources will be provided via a web portal.

The feasibility study met a number of important objectives relating to training evaluation, competency testing, adaptation and delivery of remote training and intervention which informed the development and delivery of the ROWTATE intervention RCT. It also provided valuable lessons towards remote delivery of vocational rehabilitation interventions.

This feasibility study has shown that the ROWTATE intervention and training to deliver the intervention has successfully been adapted for remote delivery. It has shown that it is feasible to deliver the remote intervention with high fidelity and attendance and that the intervention is acceptable to patients and therapists. It has also shown how to improve therapist training and competency assessment, the need for face-to-face intervention delivery where necessary and for monitoring and addressing intervention components with lower fidelity. Strategies to address these issues will be incorporated into the definitive trial.

These findings also provide valuable lessons for other similar trials providing evidence for therapist training, recruitment, feasibility of remote delivery, fidelity, attendance and acceptability of vocational rehabilitation interventions.

## Supplementary material

10.1136/bmjopen-2025-104518 online supplemental file 1

## Data Availability

Data are available on reasonable request.
