# Supplementary material for: Mixed-methods non-randomised single-arm feasibility study assessing delivery of a remote vocational rehabilitation intervention for patients with serious injury: the ROWTATE study
Source: BMJ Open. 2025 Nov 27;15(11):e104518. doi: 10.1136/bmjopen-2025-104518 (PMC12666197; doi:10.1136/bmjopen-2025-104518)
Supplement: online supplemental file 1 [file bmjopen-15-11-s001.docx]

Supplementary table 1: Details and properties of measurement tools used in the post-training questionnaire, the pre and post-adapted training questionnaire and the participant baseline questionnaire.

| **Post-training questionnaire** | |
| --- | --- |
| EBPAS | The Evidence-Based Practice Attitude Scale (EBPAS)(1, 2) assesses attitudes toward adoption of innovation and Evidence Based Practice (EBP).The 15 item version was used with 5 option responses ranging from not at all = 0 to extremely useful = 4. The EBPAS assesses four dimensions of attitudes toward adoption of EBPs including: a) intuitive appeal of EBP, b) likelihood of adopting EBP given requirements to do so, c) openness to new practices, and d) perceived divergence between research-based/academically developed interventions and current practice. Negatively worded items were reverse coded before calculating the overall score. Cronbach’s alpha for the 15 item scale has been previously found to be 0.83.(3) |
| EPIC | Evidence Based Practice Confidence Scale (EPIC)(3, 4) assesses confidence in using evidence-based practice. This is a validated measure using a 4 item scale with 11 option responses ranging from no confidence = 0% to completely confident = 100%. Four of the 11 items from the original scale were included. Questions not included were technical in nature and not applicable for the ROWTATE therapists. Test-retest reliability (Interclass correlation coefficient) for the full scale when used with occupational therapists has been previously found to be 0.92 and Cronbach’s alpha to be 0.89.(5) |
| Confidence | Confidence in providing the ROWTATE intervention was measured using a bespoke 8 item scale with 11 option responses ranging from no confidence=0% to completely confident=100%. |
| Usefulness and experiences of training | Single questions measured usefulness of training (5 option responses ranging from not very useful = 1 to extremely useful = 5), and whether training was what therapists expected (4 option responses ranging from completely =1 to not at all = 4). Experiences of training in terms of content, length and methods was measured using 8 individual items; with 5 option responses ranging from strongly disagree = 1 to strongly agree = 5. Negatively worded items were reversed scored. Participants were also asked to specify other training they would find useful with free text responses. |
| **Pre and post adapted training questionnaire** | |
| TDF | The questionnaire was informed by the Theoretical Domains Framework (TDF),(6) containing 14 previously validated behavioural determinants domains: (1) behavioural regulation, (2) beliefs about capabilities, (3) beliefs about consequences, (4) emotions, (5) environmental context and resources, (6) goals, (7) intentions, (8) knowledge, (9) memory, attention and decision processes, (10) optimism, (11) reinforcement, (12) skills, (13) social influences and (14) social-professional role and identity.(7)  The questionnaire contained 51 items spanning the 14 domains. Response options ranged from 1 = strongly disagree to 7 = strongly agree. Mean domain scores of ≤3.5 indicate substantial barriers and mean domain scores of ≥5 indicate facilitators to implementing the ROWTATE intervention. For the 93 item version, internal consistency (Cronback’s Apha) ranged from 0.68-0.93.(8) Shorter versions have previously been used and have been shown to have good internal consistency. (8-10) |
| **Participant baseline questionnaire** | |
| EQ-5D-5L | EQ-5D-5L measures self-reported health-related quality of life across five health dimensions (mobility, self-care, usual activities, pain, and anxiety/depression). Each dimension is assessed(11) with a single item question with 5 response options (e.g., mobility: 1 = I have no problems in walking about; 5 = I am unable to walk about; anxiety or depression 1 = I am not anxious or depressed, 5 = I am extremely anxious/depressed). A utility score is calculated from the five dimensions. A score of 1 represents full health, a score of 0 represents a state equivalent to dead and values less than 0 represent health states considered to be worse than dead.  Test-retest reliability has previously been found to be good (intra-class correlation coefficient ≥0.7)(12) ) |
| HADS | Depression and anxiety were measured using the Hospital Anxiety and Depression Scale (HADS(13, 14) [[Zigmon](https://link.springer.com/article/10.1186/s40814-021-00769-4#ref-CR44)d et al, 1983]) which has separate subscales for depression (HADS-D) and anxiety (HADS-A). Each subscale comprises 7 items with 4 response options scored from 0 to 3, with increasing scores representing greater symptoms. Item scores are summed to create subscale scores which range from 0-21. The cut-off for caseness on each subscale is 11. Cronbach's alpha for HADS-A has been found to vary across studies from 0.68 to 0.93 (mean 0.83) and for HADS-D from 0.67 to 0.90 (mean 0.82).(15) |
| IES-R | Post-traumatic stress was measured using the Impact of Event Scale-revised (IES-R)(16) [[Weiss](https://link.springer.com/article/10.1186/s40814-021-00769-4#ref-CR45) et al 1997]). The scale is composed of 22 items and three subscales measuring avoidance (8 items), intrusion (8 items), and hyperarousal (6 items) with response options ranging from 0 = not at all to 4 = extremely. Item scores are summed to create subscale scores. Subscale scores are summed to create a total scale score, which ranges from 0-88. Higher scores indicate higher symptoms of post-traumatic stress. The cut-off for caseness for the total scale score is >=33 .Cronbach’s alpha for the total scale score has previously been found to be 0.95.(17) |
| MoCA | The Montreal Cognitive Assessment score (MoCA) was used to assess a range of cognitive domains (Nasreddine et al. 2005).(18) The maximum score of 30 is calculated through completion of a series of tasks including visual spatial/executive category (5 points), language tasks (3 points), attention (6 points), naming (3 points), abstraction (2 points), delayed recall (5 points) and orientation (6 points). Scores range from 0-30 and lower scores indicate greater cognitive impairment. The cut-off for cognitive impairment is <26. Cronbach’s alpha has previously been found to be 0.82 in patients with a head injury.(19) |

Supplementary table 2. Search terms for the literature review

| **Qualitative literature search strategy (searched June 2020)**   1. Polytrauma$.mp. 2. Exp Multiple Trauma/ 3. (multiple adj3 (wound$ or injur$ or traum$ or casualt$)).mp. 4. Exp Brain injuries/ 5. Exp Brain concussion/ 6. Exp Brain hemorrhage, traumatic/ 7. Exp Brain injury, chronic/ 8. Exp Diffuse axonal injury/ 9. Brain injur*.ti,ab. 10. (TBI or TBIs).ti,ab. 11. (hypoxic brain damage or diffuse axonal injur* or DAI or DAIs).ti,ab. 12. Head injur*.ti,ab. 13. (brain adj2 trauma*).ti,ab. 14. (head adj2 trauma*).ti,ab. 15. Concussion.ti,ab. 16. Brain contusion.ti,ab. 17. Exp Paraplegia/ 18. Exp Quadriplegia/ 19. Exp Spinal cord injuries/ 20. Tetraplegia.mp. 21. SCI.mp. 22. Exp Paralysis/ 23. (injur* OR back OR lumbar OR cervical OR thoracic OR shoulder OR elbow OR wrist OR hand OR hip OR knee OR ankle OR foot) 24. Or/1-23 25. telemedicine/ or telemetry/ or exp videoconferencing/ or telecommunications/ or computer communication networks/ or remote consultation/ or remote sensing technology/ or exp telephone/ or electronic mail/ or exp internet/ 26. computer/ or exp microcomputer/ or minicomputer/ or exp cell phone/ or mobile application/ 27. (telemedicine or telemetry or telerehabilitation or tele-rehabilitation or telerehab or telehealth or tele-health or telehomecare or tele-homecare or telecoaching or tele-coaching or telecommunication$ or videoconference$ or video-conferenc$ or videoconsultation or video-consultation or telestroke or teleconference$ or tele-conference$ or teleconsultation or tele-consultation or telecare or ehealth or e-health).tw. 28. (telespeech or tele-speech or teleOT or tele-OT or telepractice or teletherap$).tw. 29. ((rehabilitation or therap$ or treatment or communication or consultation) adj5 (telephone$ or phone$ or video$ or internet$ or computer$ or sensor$ or modem or webcam or website$ or email)).tw. 30. ((remote$ or distance$ or distant) adj5 (rehabilitation or therap$ or treatment or physio$ or occupational therap$ or communication or consultation or care or specialist$ or monitor$ or virtual reality or virtual environment$ or technolog$)).tw. 31. ((cell$ or smart$ or mobile or android or internet or web) adj3 (comput$ or device or app$ or phone)).tw. 32. (smartphone or text-messag$ or (tablet adj3 (device$ or comput$))).tw. 33. Or/25-32 34. Qualitative research.pt,ti,ab. 35. Qualitative method*.pt,ti,ab. 36. Thematic analys$.ti,ab. 37. Observation$.ti,ab. 38. Interview*.ti,ab. 39. Focus group*.ti,ab. 40. Case stud*.pt,ti,ab. 41. Qualitative stud*.pt,ti,ab. 42. Lived experience.ti,ab. 43. Narrativ*.ti,ab. 44. qualitative systematic review* OR (systematic review AND qualitative) 45. evidence synthesis OR realist synthesis 46. qualitative AND synthesis 47. meta-synthesis* OR meta synthesis* OR metasynthesis 48. meta-ethnograph* OR metaethnograph* OR meta ethnograph* 49. meta-study OR metastudy OR meta study 50. Or/34-49 51. 24 and 33 and 50 52. Exp animals/ not humans.sh. 53. 51 not 52 |
| --- |

**Supplementary table 3: Topic guides for all interviews**

| **ROWTATE Feasibility Study Topic Guides**  **Participant semi-structured interview topic guide (Patient)**  **Multicentre Research Programme to Enhance Return to Work after Trauma (ROWTATE) – Feasibility of helping patients return to work during the Covid-19 pandemic**  **Chief Investigators: Dr Kate Radford & Professor Denise Kendrick**  **Over the past few months you have been receiving help/advice/support from an occupational therapist (and clinical psychologist if relevant) to help you return to work as part of the ROWTATE study. These questions are about the help/advice/support you have received:**  **General:**   - Can you start by telling us about your experience of the help/advice/support you have received as part of ROWTATE - Can you tell us about your experience of your employer taking part in ROWTATE? - Can you tell us about your experience of working with your OT/CP?   **Theoretical Framework of Acceptability:**   1. Affective attitude  - How did you/do you feel about the help/advice/support you received from the OT (and CP if relevant)?  1. Burden  - How much of an effort is/was it for you to engage with the help/advice/support you received?  1. Ethicality  - Was there any help/advice/support that didn’t feel right to you or that you disagreed with? - Did the help/advice/support have any negative consequences for you?  1. Intervention coherence  - What help/advice/support have you received for the OT (and CP if relevant)? - What do you think the help/advice/support is trying to achieve?   (Holly Walton qu: how easy has it been to act on the advice/support you have been given?)   1. Opportunity costs  - What has it cost you in terms of money, time, other resources to engage with the help/advice/support from the OT (and CP if relevant)?  1. Perceived effectiveness  - How well do you think the help/advice/support is working/has worked for you?  1. Self-efficacy   How confident are you that you can act on the help/advice/support from the OT (and CP if relevant)?  What was that advice and why was it difficult?  **Remote delivery of the intervention:**   - What do you think about getting the help/advice/support from the OT (and CP if relevant) via phone/videocalls/other remote methods? - What were the challenges to getting the help/advice/support in this way? - How do you think getting help/advice/support by phone/videocall etc affected your relationship with your OT/CP? - Overall, how well did the technology work for you?   If experienced any difficulty with technology:   - - What difficulties did you encounter and how could they be overcome in the future? - Was there any technology/devices/software that would have helped but didn’t have? - If you had a choice in future to have help/advice/support provided face-face or by phone/videocalls/other remote methods, which would you prefer and why? - Is there anything else you’d like to tell us about the help/advice/support you received by phone/videocall/other remote methods?   **COVID specific questions**   1. **Prior to the Covid-19 pandemic, had you returned to work?**   ***If no***   - *Can you tell me about why you hadn’t returned to work?* - *Did anyone suggest any alternative options (e.g., new job role)?*     ***If yes***   - - *How long had you been back at work?*   - *Phased return, part time, full time? Paid? Voluntary?*   - *What was helpful in the RTW process?*   - *What was unhelpful in the RTW process?*   - *How had work changed since your stroke? Did you have any adjustments or adaptations put in place? (e.g., changes to role/responsibilities, use of specialist equipment, use of energy conservation techniques, changes in how they travel to work)*   - *Had anything gone particularly well since you had been back at work?*   - *Had you experienced any difficulties since you had been back at work?*   - *Had/have you received any support since returning to work? (health professional, employer, family, etc)*   ***If yes***  o *What did they do to support you? (e.g., give information, advice, suggestions)*  o *(if discussing health professonal) Did they provide any support to your family or employer?*  o *Is there anything else they could have done to help you or your family/employer?*  **2. Has the Covid-19 pandemic affected your return to work in any way in any way?**   - - *Contact/support from others (health professionals, employer)*   - *Access to resources/equipment (e.g., computer)*   - *Transport to/from workplace*   - *Returning to work sooner than ideal (e.g., essential worker, needed money)*   - *Started new temporary role (e.g., supermarket, NHS volunteer, childcare)*   - *Reduced hours, or not able to work (graded return postponed, furloughed, shielding/self-isolating, made redundant)*     **3. Have you experienced any unexpected benefits in how you’ve been treated during the Covid-19 pandemic?**   - - *More regular contact with health professionals/employers*   - *Provision of support (e.g., information, advice, equipment, financial)*   - *Reduction in fatigue (e.g., reduced travel time etc)*   Prompts:   - * Tell me more about that … - * How did that feel? - * Can you give me an example of … - * What do you mean by … |
| --- |
| **ROWTATE Feasibility Study Topic Guides**  **Participant semi-structured interview topic guide (Therapists pre-intervention)**  **Multicentre Research Programme to Enhance Return to Work after Trauma (ROWTATE) – Feasibility of helping patients return to work during the Covid-19 pandemic.**  **(Chief Investigators: Professor Denise Kendrick and Dr Kate Radford)**  **General:**   - Have you had to adapt clinical practice during COVID19 to deliver services remotely? If so, how? - What devices and online platforms have you been using? - Have you experienced any issues in delivering tele-rehabilitation or telepsychology? - What are the main factors that could help or hinder the delivery of ROWTATE via tele-rehabilitation? - What additional support might you require?   **Knowledge**   - What experience do you have of delivering tele-rehabilitation or telepsychology? - Have you had any formal or informal training in doing this?   - Via professional organisation, NHS Trust, online (e.g. YouTube) - Are there any gaps in your knowledge?   **Social/professional role and identity**   - To what extent is tele-rehabilitation or telepsychology part of your professional role?   **Skills**   - How easy or difficult would you find delivering the ROWTATE intervention via tele-rehabilitation or telepsychology? - Do you think there are any particular skills required to do this? - What additional training would you need? - Are there any aspects of vocational rehabilitation that you might find difficult to deliver remotely?   - Workability assessments?   - Cognitive assessments?   - Work hardening?   - Work site assessments?   **Beliefs about capabilities**   - How confident are you that you could deliver the ROWTATE intervention via tele-rehabilitation or telepsychology?   If not very confident:   - Is there any specific training that could help increase your confidence?   **Beliefs about consequences**   - Do you think there would be any benefits or disadvantages of delivering the ROWTATE intervention via tele-rehabilitation or telepsychology?   **Environmental context and resources**   - What work factors or resources may help or hinder the delivery of ROWTATE via tele-rehabilitation or telepsychology?   - Access to work phone, cost of calls, free Wifi, security issues   - Support from NHS Trust/manager/IT support   - Hardware/software/licences - Are there systems in place at work to support remote delivery of the intervention?   - NHS Trust policies/guidelines   - Training   - Access to the internet   **Training**   - Have you participated in any form of online training?   If yes, what platforms did this use?   - Zoom, MS Teams, Skype, Adobe connect, other - What training do you think you would need to provide the ROWTATE intervention remotely?   - Training in using devices and/or software for remote delivery?   - Training in online security/data protection/online safety?   - Training in providing specific aspects of vocational rehabilitation remotely e.g: workability assessments, cognitive assessments, work hardening, work site assessments   - Any other training needs you can think of?   Thinking now about the ROWTATE training you received in January:   - How do you think we can adapt the ROWTATE training you received to be able to provide it remotely in the future? - Assuming the ROWTATE training can be adapted and delivered online using a combination of lectures and interactive workshops – what would be your preference for delivery format?   e.g. 4 half day training sessions with breaks?  A series of brief (1-2 hour) daily/ bi-weekly training sessions over a 1-2 week period  What times of day would work best for you e.g. early morning 8.30-10, mid morning10.30-12, lunchtimes 12-1.30 afternoon 1.30-3, evenings e.g. 4-6pm   - From your memory of the ROWTATE training, which aspects of the training do you think would work well remotely?   - Format such as group work, presentations, discussions   - Working with PPI, meeting people from other centres, meeting study team, sharing knowledge - Which aspects of the training might not work so well remotely?   - Same prompts as above - In the ROWATE training we had an actor play the part of a patient and you completed individual and team tasks. Assuming we can create an online version of the team based assessment (TOSCE), how do you think we could do the individual and team tasks remotely? - What online platforms would you prefer the training to be provided using and why? - Can you foresee any technological difficulties for you if the training was provided remotely? - Is there anything else you would like to tell us about adapting the ROWTATE training? |
| **ROWTATE Feasibility Study Topic Guides**  **Participant semi-structured interview topic guide (Therapists: post-intervention)**  Multicentre Research Programme to Enhance Return to Work after Trauma (ROWTATE) – Feasibility of helping patients return to work during the Covid-19 pandemic  **(Chief Investigators: Professor Denise Kendrick and Dr Kate Radford)**  **General:**   - Tell us about your experience of providing help/advice/support to patients and their employers during ROWTATE - What did you find worked best? - What did you find did not work that well? - If you could make changes to the help/advice/support in ROWTATE, what would you change and why?   **Theoretical Framework of Acceptability:**   1. Affective attitude  - How do you feel about the help/advice/support you have provided during ROWTATE?  1. Burden  - How much of an effort was it for you to provide the help/advice/support during ROWTATE?  1. Ethicality  - To what extent does/did the help/advice/support you provided during ROWTATE feel right to you or fit with the values of your employing organisation? - Did providing the ROWTATE help/advice/support have any negative consequences for you, your patients or their employers?  1. Intervention coherence  - What support/help/advice have you provided for patients and employers during ROWTATE? - What is/was the help/advice/support you provide in ROWTATE trying to achieve?   (Holly Walton qu fidelity: how well do you think patients have been able to act on your advice?)   1. Opportunity costs  - What has it cost you in terms of money, time, other resources to provide ROWTATE help/advice/support?  1. Perceived effectiveness  - How well do you think ROWTATE help/advice/support is working/has worked?  1. Self-efficacy  - How confident are you that you can provide ROWTATE help/advice/support to patients and their employers?   **Remote delivery of the intervention:**   - What do you think about providing the ROWTATE help/advice/support via phone/videocalls/other remote methods?   - What challenges did find in doing this? - Are there any groups of patients that you think remote help/advice/support is less suitable for?   - Why is this?   - How might difficulties with remote provision with these patients be overcome in the future? - How do you think providing help/advice/support remotely affected your relationship with your patient?   If any negative impacts mentioned:   - - How do you think this affected how well your patient engaged with the help/advice/support? - Did you provide any help/advice/support face-face with patients or employers?   - Why did you choose face-face for this help/advice/support? - Overall, how well did the technology work for providing help/advice/support remotely?   If experienced any difficulty with technology:   - - What difficulties did you encounter and how could they be overcome in the future? - How well did the ROWTATE training prepare you to provide help/advice/support for patients and employers remotely? - If you had a choice in future to provide the ROWTATE help/advice/support face-face or by phone/videocalls/other remote methods, which would you prefer and why? - Is there anything else you would like to tell us about your experiences of providing the ROWTATE help/advice/support remotely?   **COVID specific questions**  **1. Do you feel that the Covid-19 pandemic has impacted on your patient’s ability to return to work?**   - How has it impacted on return to work? - At what point the OT/CP decides the patient is ready for discharge if they are furloughed as there is no job to return to at that point? - What about patients that have had to shield, self-isolate or contracted Covid-19?  What impact has this had on their return to work? - What experience do they have of patients losing their jobs because of COVID?   - How has this affected the intervention they provided? - Has their employer been supportive? If not, have you managed to overcome challenges and how?   **2. Do you feel that the Covid-19 pandemic has impacted on patients in other ways?**   - Are patients requiring more psychological support during their recovery and return to work? Do you think this is Covid related? - Are patients experiencing more financial challenges? - Any other effects of Covid that have impacted on someone’s ability return to work?   **3**.  **Has the Covid-19 pandemic affected your ability to deliver the intervention in any way?**   - - Ability to contact necessary stakeholders without needing to book face-to-face appointment/more flexible (e.g. patients, employers)   - Access to resources/equipment (e.g., computer to deliver sessions)   - Challenges with assessing patients remotely, risks associated with face-to-face sessions   - Employers having to furlough staff, not being supportive, patients having to self-isolate etc.     **4. Have you experienced any unexpected benefits in delivering the intervention during the Covid-19 pandemic?**   - - Ability to have more regular and flexible contact with patients/employers   - Reduction in time spent doing additional non-therapy activities (e.g., reduced travel time etc)   - Patients not feeling anxious about attending face-to-face appointments, patients not having to travel to appointments so reduced fatigue etc.   Prompts:  * Tell me more about that …  * How did that feel?  * Can you give me an example of …  * What do you mean by … |

Supplementary table 4. Criteria for progression from the feasibility study to the main trial (progression: Green=proceed. Amber=review and adapt intervention/processes. Red=stop).

| **Study Objective** | **Measurement criteria** | **Method of measurement** | **Achievement of criteria** |
| --- | --- | --- | --- |
| 1. Adapt the ROWTATE intervention to make it suitable for remote delivery, as much as possible, via tele-rehabilitation and tele-psychology | Green: ≥60% of OT-patient and CP-patient contacts are delivered remotely (including video-call, phone, email, messaging)  Amber: 40-59% of contacts are delivered remotely  Red: <40% of contacts are delivered remotely  Where contacts are not delivered remotely, reasons for this will be identified enabling, where possible, strategies for improvement to be identified. | Intervention CRFs recorded mode of delivery of each contact and reasons for any face-face contacts  Mentoring records recorded information on which contacts are being delivered face-face and why. | Green  98.5% of OT and 100% of CP contacts were delivered remotely.  Reasons for face-face contacts identified and strategies for improvement identified. . |
| 2. Adapt the ROWTATE OT and CP training to make it suitable for remote delivery | Feasibility study training:  Green:≥70% of OTs and CPs felt the training prepared them sufficiently to deliver the remote intervention  Amber: 50-69% of OTs and CPs felt the training prepared them sufficiently to deliver the remote intervention  Red: <50% of OTs and CPs felt the training prepared them sufficiently to deliver the remote intervention  Where OTs and CPs did not feel the training prepared them sufficiently, reasons for this will be identified enabling, where possible, strategies for improvement to be identified. | Therapist post intervention interviews | Green  100% of OTs and CPs felt the training prepared them sufficiently to deliver the remote intervention. |
| 3. Deliver the adapted ROWTATE intervention and assess feasibility and fidelity of remote delivery via tele-rehabilitation and tele-psychology | Green: ≥90% of participants commence the intervention within the specified time period (i.e. 0-12 weeks post-injury)  Amber: 60-89% of participants commence the intervention within the specified time period  Red: <60% of participants commence the intervention within the specified time period  Where the intervention is not commenced within the specified time frame, reasons for this will be identified enabling, where possible, strategies for improvement to be identified. | Intervention CRFs recorded date of initial session  Mentoring records recorded information on reasons for not commencing intervention | Green  90% of participants commenced the intervention within 12 weeks of injury.  Reasons for not commencing intervention and strategies for improvement identified. |
|  | Green: ≤30% withdraw from the intervention before the agreed end date  Amber: 31-50% withdraw from the intervention before the agreed end date  Red: >50% withdraw from the intervention before the agreed end date  Where withdrawals before the agreed end date occur, reasons for withdrawal will be identified, enabling, where possible, strategies for improvement to be identified. | Intervention CRFs recorded date of withdrawal  Mentoring records recorded information on reasons for withdrawal | Green  0% of participants withdrew from the intervention |
|  | Core components of the intervention are identified  Extent to which core components are delivered is measured  Factors affecting delivery of core components are identified | Intervention CRFs recorded each component of the intervention  Fidelity checklist recorded delivery of core components  Mentoring records recorded factors affecting delivery of core components | Core intervention components identified and measured. Factors affecting delivery of core components identified. |
| 4. Assess acceptability, barriers and facilitators to remote delivery of the ROWTATE intervention via tele-rehabilitation and tele-psychology | Acceptability is assessed across the 7 domains in the Theoretical Framework of Acceptability and where possible strategies for increasing acceptability are identified  Barriers and facilitators to remote delivery of the intervention are identified and where possible strategies to enhance facilitators and reduce barriers are identified. | Patient, therapist and employer post intervention interviews | Intervention was acceptable to patients and therapists. Unable to conduct employer interviews due to participants being self-employed or not providing consent for employers to be contacted or employers not responding to invitations. (Full details published elsewhere).(20) Strategies to enhance employer engagement in the main trial have been identified.  Barriers and facilitators to remote delivery identified and strategies to enhance facilitators and reduce barriers are identified. |

Supplementary table 5. Description of the adapted intervention based on the TIDiER checklist

|  | **Description** |
| --- | --- |
| **Brief Name** (Provide the name or a phrase that describes the intervention.) | ROWTATE – Return to work after trauma |
| **WHY** Describe any rationale, theory, or goal of the elements essential to the intervention. | **Goal**   - ROWTATE is a 12-month case coordinated job/education retention intervention involving occupational therapy support with vocational goal setting, provision of workplace accommodations, communication with employers, advice for the patient’s family and employer, identification of mental health issues and exploration of workplace alternatives as required. - Clinical psychologists (CPs) provide input to patients experiencing mental health problems, following a stepped care approach. - The intervention explores alternatives to pre-injury employment in cases where return to pre-existing employer is not feasible or is unsustainable.   **Underpinning Theory**  The intervention is underpinned by the International Classification of Functioning, Disability and Health (ICF), a biopsychosocial framework that considers the overall context of an individual. Importantly it takes into account the interactions between environmental (e.g., the workplace) and injury related variables, recognising that work disability is created or removed as a result of the interaction between biological, psychological, and social factors. As such, the intervention focuses on modifying work tasks and removing environmental barriers in addition to restorative approaches to promote functional recovery and psychological adjustment. We also draw on the ‘Work Disability Arena’ or Sherbrooke model (Loisel et al., 2005) which considers the different systems (personal, workplace, healthcare and compensation system) that surround the worker, and influence return-to-work (RTW). Hence, ROWTATE adopts a case-coordinated approach to cross discipline, cross system and cross sector communication. The ROWTATE occupational therapist (OT) takes on the role of case manager. |
| **WHAT**  **Materials**: Describe any physical or informational materials used in the intervention, including those provided to participants or used in intervention delivery or in training of intervention providers. Provide information on where the materials can be accessed (e.g. online appendix, URL).  **Procedures**: Describe each of the procedures, activities, and/or processes used in the intervention, including any enabling or support activities. | During the COVID-19 pandemic, our original face-to-face intervention had to be adapted for remote delivery. The adapted intervention had the same content as the original intervention, but was to be delivered predominantly via video or phone call or phone.  The adaptations were informed by collaboration with the Brain Recovery and Rehabilitation Research Group at Monash University, Australia. Adaptation focussed on components that would typically be delivered face-to-face (e.g. workplace assessment, work hardening). We developed telerehabilitation specific work hardening templates, assessment forms and provided examples for conducting remote workplace assessments. The collaboration also informed the OT/CP training, to ensure therapist understanding of remote intervention delivery  **Materials:**   - OTs and CPs given ROWTATE intervention manual detailing the intervention content, rationale and objectives, examples of RTW plans and useful resources. - OTs/CPs were provided with a laptop to conduct the remote sessions with study participants.   **Procedures:**  ***Training therapists to deliver the intervention***  The training package comprised a 2-day face-to-face taught programme delivered by the ROWTATE training team, supported by monthly mentoring from a vocational rehabilitation expert OT and CP (members of the training team).  Additional training in delivering telerehabilitation was added in September 2020 to teach therapists how to adapt the ‘work hardening’ ‘workability assessment’ and ‘worksite assessment’ elements of the intervention for delivery by tele-rehabilitation. This was delivered remotely on MS Teams. Additional “how to set up/conduct a telerehabilitation session” resources were provided to therapists that related to the core components of the intervention.  ***Delivery of the intervention***  ROWTATE involves an OT working in a case coordinator role with a wider team of healthcare professionals, employers, family members and other agencies (e.g., solicitors, insurance and employment agencies) to:   - Assess the impact of the injury on the participant, family and the patient’s role as a worker/student and their ability to do their job/educational course. - Educate participants, employers/tutors and families about the effects of the injury and its impact on work/education and find acceptable strategies to lessen the impact. - Continually monitor and assess the patient’s post-injury life and work/educational goals. - Prepare people for work/education by establishing structured routines with gradually increased activity levels and opportunity to practice work/study skills, e.g., structured computerised cognitive stimulation to increase concentration, daily walks to increase physical stamina. - Liaise with employers/tutors, employment advisors, student services, solicitors and the healthcare team to advise about the effects of the injury and to plan and monitor a phased RTW.   During initial assessment and at 6 months post-injury, the OT screens participants for mental health problems using standardised measures of mood, depression and post-traumatic stress disorder (PTSD) (using GAD-2, Whooley Depression questions, Hospital Anxiety and Depression Scale, Impact of Event Scale, PHQ Panic Disorder Questionnaire). If patients score within the ‘case’ or ‘borderline’ threshold for any of the measures, they are discussed with or referred to CP for further assessment.  Following CP assessment participants either receive: 1) no CP intervention, 2) monitored by the OT for further mental health issues, 3) seen by the CP or 4) referred to other local psychological or mental health services.  Psychological interventions are delivered 1 to 1 and include evidence-based approaches for managing trauma-related mental health issues such as anxiety, depression and PTSD, assessment of the impact of mental health problems on work ability, teaching coping strategies, e.g., fatigue and anxiety management for use in the workplace. |
| **WHO PROVIDED** | **Intervention provider qualifications**  HCPC registered occupational therapist (OT) (BSc OT) and where needed, assessment and management of psychological problems by a qualified (DClinPsy) and HCPC registered clinical psychologist (CP)  **Intervention provider background and experience**  OTs with experience of working with people with serious/traumatic injuries and vocational rehabilitation (desirable)  CPs with experience of delivering interventions to manage anxiety, depression and PTSD following trauma (e.g., trauma-focused CBT).  **Specific training provided**  2-day face-to-face VR training session, plus two additional half-days of telerehabilitation training, provided by ROWTATE training team. This comprised an academic occupational therapist with extensive experience in delivering vocational rehabilitation, supported by members of the research team with expertise in vocational rehabilitation, long-term conditions, trauma, psychology and implementation.  **Resources**  Workplace accommodations from the Job Accommodations Network (JAN) <https://askjan.org/>  Goal Attainment Scaling (GAS) <https://www.kcl.ac.uk/cicelysaunders/resources/tools/gas> |
| **HOW** | **Mode of delivery**   - OT delivered 90% remotely via telerehabilitation (video call or phone call) on a 1 to 1 basis. Remote sessions typically last an hour. - CP delivered 100% remotely 1 to 1. In 10% of cases, CP and OT liaise directly to formulate a RTW plan. In some cases, they work together with the employer to plan RTW.   **Other**  Additional time spent in liaison (letters, phone and video calls) with the patient, employer, family or other healthcare providers and employment stakeholders. |
| **WHERE** | **Where provided**  Intervention delivered in the community (at home or in the workplace) via telerehabilitation (via phone, video call, email, etc.) where possible. Where necessary, the OT or CP visit the patient’s home or workplace should additional, and essential, in-person assessments be required.  Patients still in hospital or in a rehabilitation unit when the intervention begins may be seen in person or remotely.  **Necessary infrastructure**   - Access to laptops and approved delivery platform (e.g Cisco WebEX, DrDoctor, Attend Anywhere) for OTs and CPs - Telephone, smartphone, laptop or iPad/tablet for patients |
| **WHEN and HOW MUCH** Describe the number of times the intervention was delivered and over what period of time including the number of sessions, their schedule, and their duration, intensity or dose. | **Intervention delivery time**  Intervention commences within 12 weeks of injury and continues for up to 12 months post randomisation. Duration and frequency tailored to individual need.  **Number of sessions and length**   - Each intervention session will last for approximately 1 hour. - Average number of sessions per participant estimated at 6. - The length of intervention will not extend beyond 12 months.   **Frequency of sessions**   - Participants received the OT intervention for a median of 34.6 weeks. - OTs delivered a median of 14.25 hours of intervention per participant during a median of 13 sessions (IQR 9.5, 14) per participant - Most sessions were delivered early in the intervention, with the highest frequency of OT sessions per participant in month 2. - Participants received the CP intervention for a median of 7.9 weeks. - CPs delivered a median of 3.5 hours of intervention per participant during a median of 3 (IQR 1,8.5) sessions per participant. - The highest frequency of CP sessions per participant were delivered in month 1. Three participants received only one CP session. |
| **TAILORING** | The OT and psychological interventions will be tailored in duration and frequency according to individual need over a 12-month period. |
| **MODIFICATIONS** If the intervention was modified during the course of the study, describe the changes (what, why, when, and how). | Prior to starting the intervention delivery in October 2020, the intervention was adapted from face-to-face delivery to remote delivery as a result of the COVID-19 pandemic.  The intervention is designed to be individually tailored for each individual, but no modifications were made during the course of the study. |
| **HOW WELL** Planned: If intervention adherence or fidelity was assessed, describe how and by whom, and if any strategies were used to maintain or improve fidelity, describe them. | **Planned**  Monthly mentoring sessions with OTs/CPs led by experienced VR OT and clinical neuropsychologist to identify implementation and fidelity issues, and discuss adherence to protocol and addressing implementation barriers and contextual and process issues related to intervention delivery. Measured by completion of intervention delivery case report forms (CRFs) and completion of mentoring records 0-12 months post-participant recruitment.  Fidelity assessed by checklist and quantitatively using content CRFs, qualitative data from intervention and mentoring records and interviews used to identify those factors that moderate fidelity. |
| Actual: If intervention adherence or fidelity was assessed, describe the extent to which the intervention was delivered as planned. | **Actual**   - A high percentage of core and desirable intervention components were delivered as planned (84.5% for OTs and 92.9% for CPs). - The intervention stage with the lowest fidelity was the discharge process stage (57.1% for OTs and 75.0% for CPs). - See Table 5 for more detail. |

References

1. Aarons GA. Mental Health Provider Attitudes Toward Adoption of Evidence-Based Practice: The Evidence-Based Practice Attitude Scale (EBPAS). Mental Health Services Research. 2004;6(2):61-74.

2. Aarons GA. Confirmatory factor analysis of the Evidence-Based Practice Attitude Scale in a geographically diverse sample of community mental health providers. Adm Policy Ment Health. 2007;34.

3. Ayhan Başer D, Ağadayi E, Gönderen Çakmak S, Kahveci R. Adaptation of the evidence-based practices attitude scale-15 in Turkish family medicine residents. International journal of clinical practice. 2021;75(8):e14354.

4. Salbach NM, Jaglal SB. Creation and validation of the evidence-based practice confidence scale for health care professionals. Journal of Evaluation in Clinical Practice. 2011;17(4):794-800.

5. Clyde JH, Brooks D, Cameron JI, Salbach NM. Validation of the Evidence-Based Practice Confidence (EPIC) Scale With Occupational Therapists. The American journal of occupational therapy : official publication of the American Occupational Therapy Association. 2016;70(2):7002280010p1-9.

6. Michie S, Johnston M, Abraham C, Lawton R, Parker D, Walker A. Making psychological theory useful for implementing evidence based practice: a consensus approach. Quality & safety in health care. 2005;14(1):26-33.

7. Cane J, O’Connor D, Michie S. Validation of the theoretical domains framework for use in behaviour change and implementation research. Implementation Science. 2012;7(1):37.

8. Huijg JM, Gebhardt WA, Dusseldorp E, Verheijden MW, van der Zouwe N, Middelkoop BJC, et al. Measuring determinants of implementation behavior: psychometric properties of a questionnaire based on the theoretical domains framework. Implementation Science. 2014;9(1):33.

9. Smith JD, Corace KM, MacDonald TK, Fabrigar LR, Saedi A, Chaplin A, et al. Application of the Theoretical Domains Framework to identify factors that influence hand hygiene compliance in long-term care. J Hosp Infect. 2019;101(4):393-8.

10. MacCallum L, Mathers A, Kellar J, Rousse-Grossman J, Moore J, Lewis GF, et al. Pharmacists report lack of reinforcement and the work environment as the biggest barriers to routine monitoring and follow-up for people with diabetes: A survey of community pharmacists. Research in Social and Administrative Pharmacy. 2021;17(2):332-43.

11. Herdman M, Gudex C, Lloyd A, Janssen M, Kind P, Parkin D, et al. Development and preliminary testing of the new five-level version of EQ-5D (EQ-5D-5L). Qual Life Res. 2011;20(10):1727-36.

12. Feng YS, Kohlmann T, Janssen MF, Buchholz I. Psychometric properties of the EQ-5D-5L: a systematic review of the literature. Qual Life Res. 2021;30(3):647-73.

13. Zador P. Adoption of right turn on red: effects on crashes at signalized intersections. Accid Anal Prev. 1982;14(3):219-34.

14. Zigmond AS, Snaith, R.P. The Hospital Anxiety and Depression Scale. Acta Psychiatrica Scandinavica. 1983;67(6):361-70.

15. Bjelland I, Dahl AA, Haug TT, Neckelmann D. The validity of the Hospital Anxiety and Depression Scale: An updated literature review. Journal of Psychosomatic Research. 2002;52(2):69-77.

16. Weiss DS, Marmar CR. The Impact of Event Scale—Revised. Assessing psychological trauma and PTSD. New York, NY, US: Guilford Press; 1997. p. 399-411.

17. Beck JG, Grant DM, Read JP, Clapp JD, Coffey SF, Miller LM, et al. The impact of event scale-revised: psychometric properties in a sample of motor vehicle accident survivors. Journal of anxiety disorders. 2008;22(2):187-98.

18. Nasreddine ZS, Phillips NA, Bédirian V, Charbonneau S, Whitehead V, Collin I, et al. The Montreal Cognitive Assessment, MoCA: a brief screening tool for mild cognitive impairment. j Am Geriatr Soc. 2005;53(4):695-9.

19. Wong GKC, Ngai K, Lam SW, Wong A, Mok V, Poon WS. Validity of the Montreal Cognitive Assessment for traumatic brain injury patients with intracranial haemorrhage. Brain Inj. 2013;27(4):394-8.

20. Kettlewell J, Lindley R, Radford K, Patel P, Bridger K, Kellezi B, et al. Factors Affecting the Delivery and Acceptability of the ROWTATE Telehealth Vocational Rehabilitation Intervention for Traumatic Injury Survivors: A Mixed-Methods Study. Int J Environ Res Public Health. 2021;18(18).
