# Supplementary material for: Mixed-methods non-randomised single-arm feasibility study assessing delivery of a remote vocational rehabilitation intervention for patients with serious injury: the ROWTATE study
Source: BMJ Open. 2025 Nov 27;15(11):e104518. doi: 10.1136/bmjopen-2025-104518 (PMC12666197; doi:10.1136/bmjopen-2025-104518)
Supplement: Supplementary file 2 [file bmjopen-2025-104518.reviewer_comments.pdf]

PEER REVIEW HISTORY

BMJ Open publishes all reviews undertaken for accepted manuscripts. Reviewers are asked to complete a checklist review form and are provided with free text boxes to elaborate on their assessment. These free text comments are reproduced below.

ARTICLE DETAILS

Title (Provisional)

Mixed methods non-randomised single-arm feasibility study assessing delivery of a remote vocational rehabilitation intervention for patients with serious injury: the ROWTATE study

Authors

Kellezi, Blerina; Holmes, Jain; Kettlewell, Jade; Lindley, Rebecca; Radford, Kate; Patel, Priya; Bridger, Kay; Lannin, Natasha A; Andrews, Isobel; Blackburn, Lauren; Brooks, Adam; das Nair, Roshan; Fallon, Steve; Farrin, Amanda; Hoffman, K; Jones, Trevor; Morriss, Richard; Timmons, Stephen; Kendrick, Denise

---

VERSION 1 - REVIEW

**Reviewer**	**1**
**Name**	**Sim, Alison**
**Affiliation**	**University of Sydney, Faculty of Medicine and Health**
**Date**	**11-Aug-2025**
**COI**	**None**

Summary feedback: This is a very thorough feasibility study that has been undertaken prior to the implementation of a larger RCT. The study has an emphasis on the training and support of the clinicians delivering the intervention. It also presents a strong focus on fidelity and the remediations that followed the identification of issues that were likely to be important for the larger trial. Considerable care has been taken to document the processes undertaken to deliver both the training and intervention including the pivots that were required with the onset of Covid.

I recommend the following minor edits:

There is a typo – Page 7(as per page numbers on top left or top ride side – page number of proof) , Line 8, Patients are *Screened*

Page 10, Line 54 – Could you provide a reference for the intervention trials that were delivered in Australia? If they are not yet published, could you adjust the wording of this section to reflect this?

Page 12, Invited to participate via post, telephone or in-person ~~to participate~~

Comment: The methodology of the therapist training appears to be very comprehensive despite the challenges of COVID. It clearly aims to enhance fidelity of the intervention.

Page 18, Line35/36 Comma missing after the word 'narrow'

Page 20, Line 11. It would be helpful to have included the interview guide as a supplement

Page 21, Line 33, and Page 22, Line 31 It would be good to have a reference for the use of framework analysis.

Page 23, Line 12: The sentence starting with: The lowest score….. does not make sense. It could either mean that therapists had questions and uncertainties or had no unanswered question. Could you alter the language so that it is less ambiguous?

Page 28, Line 29/31 : I think you meant to say "preferable FOR the main trial"?

Page 29, Line 56 -59 –this section "give criterion here" x 3 needs to be addressed.

Discussion:

Page 35, Line 10

Comparisons with previous research

Given that you worked with the Australian researchers (ref 49) to develop the intervention, is it worth reflecting on the comparison between your outcomes and experiences and their paper(s) in this section?

Reviewer 2

Name **Rossettini, Giacomo**

Affiliation **University of Genoa, Neuroscience, Rehabilitation, Ophthalmology, Genetics, Maternal and Child Health**

Date **12-Aug-2025**

COI **None**

Dear Authors,

Thanks a lot for the opportunity you have offered me to revise the manuscript “Mixed methods non-randomised single-arm feasibility study assessing delivery of a remote vocational rehabilitation intervention for patients with serious injury: the ROWTATE study". I thank the authors for their efforts in producing this study. It perfectly aligns with my research and expertise; thus, I am confident I can offer a valuable peer review.

As a significant strength, this paper evaluates therapists’ training and competence, adapts the intervention and training for remote delivery and assesses feasibility and fidelity of remote delivery to inform a definitive randomised controlled trial.

As a weakness, the manuscript presents a few issues and needs more details and clarity concerning methodological elements, results presentation and discussion that are useful for understanding its content.

Therefore, my overall peer review judgment is a major revision. In this phase I have suggested macro points for improvement. In the next phase, I will provide more other details I hope to see the revised paper soon.

¶AREAS OF IMPROVEMENT

#GENERAL:

*abbreviations: please report ALL the abbreviations in full in the whole manuscript.

*English: please revise the English of the manuscript

#TABLES and FIGURES:

*abbreviations: please report ALL the abbreviations in full in a legend (also in the Supplementary file).

*add a figure to sinthetise the findings

#ABSTRACT:

*keywords: please check if the keywords were MeSh terms.

#INTRODUCTION

* background and rationale: Please strengthen the value of remote training to promote education to healthcare providers. A useful reference could be this one (doi: 10.1007/s40670-021-01439-z).

#METHODS and RESULTS:

*Reporting and analysis: it is a mixed methods, but there is no evidence of the use of frameworks shared by the scientific community (e.g., Creswell and Clark) that considers sequence, priority and integration of findings and their transformation gradient (QUANT-?QUAL or QUAL ? QUANT) [Creswell JW, Clark VL. Designing and conducting mixed methods research. 3rd ed. Thousand Oaks, CA, USA: Sage Publications, 2018]. In addition, the Good Reporting of a Mixed Methods Study (GRAMMS) criteria for mixed methods was not used [O'Cathain A, Murphy E, Nicholl J. The quality of mixed methods studies in health services research. J Health Serv Res Policy 2008; 13: 92-98]. Please revise and organize accordingly

#DISCUSSION

*reporting: please organize the discussion as main findings, comparison with evidence, strengths and weakness, possible implications for clinical practice and research. I suggest adding the implications.

VERSION 1 - AUTHOR RESPONSE

**Reviewer 1's comments**	
This is a very thorough feasibility study that has been undertaken prior to the implementation of a larger RCT. The study has an emphasis on the training and support of the clinicians delivering the intervention. It also presents a strong focus on fidelity and the remediations that followed the identification of issues that were likely to be important for the larger trial. Considerable care has been taken to document the processes undertaken to deliver both the training and intervention including the pivots that were required with the onset of Covid.	We thank the reviewer for these positive comments.
There is a typo – Page 7 (as per page numbers on top left or top ride side – page number of proof), Line 8, Patients are *Screened*	Thank you. We have now corrected this.
Page 10, Line 54 – Could you provide a reference for the intervention trials that were delivered in Australia? If they are not yet published, could you adjust the wording of this section to reflect this?	We have made reference to this:<br><br>Cox NS, Scrivener K, Holland AE, Jolliffe L, Wighton A, Nelson S, et al. A Brief Intervention to Support Implementation of Telerehabilitation by Community Rehabilitation Services During COVID-19: A Feasibility Study. Arch Phys Med Rehabil. 2021;102(4):789-95.
Page 12, Invited to participate via post, telephone or in-person ~~to participate~~	Thank you. We have now corrected this.
Comment: The methodology of the therapist training appears to be very comprehensive despite the challenges of COVID. It clearly aims to enhance fidelity of the intervention.	We thank the reviewer for this positive comment.
Page 18, Line35/36 Comma missing after the word 'narrow'	Thank you. We have now corrected this.

Page 20, Line 11. It would be helpful to have included the interview guide as a supplement	All three interview guides have been added as a supplementary file 3.
Page 21, Line 33, and Page 22, Line 31 It would be good to have a reference for the use of framework analysis.	We have now added a reference to framework analysis (Gale at al, 2012) in both sections.
Page 23, Line 12: The sentence starting with: The lowest score….. does not make sense. It could either mean that therapists had questions and uncertainties or had no unanswered question. Could you alter the language so that it is less ambiguous?	Thank you for pointing out the lack of clarity in this sentence. We have rephrased it. It now reads<br>"The lowest score was for having unanswered questions or uncertainties at the end of the training, indicating that there was a low level of agreement with this statement and most had their questions answered."
Page 28, Line 29/31 : I think you meant to say "preferable FOR the main trial"?	This has now been corrected.
Page 29, Line 56 -59 –this section "give criterion here" x 3 needs to be addressed.	Thank you for pointing this out. All three criteria have now been added.
Page 35, Line 10<br>Comparisons with previous research<br>Given that you worked with the Australian researchers (ref 49) to develop the intervention, is it worth reflecting on the comparison between your outcomes and experiences and their paper(s) in this section?	We have amended the text to include a comparison with the Australia study (ref 49). The text now reads:<br><br>The barriers and facilitators to remote delivery of the intervention identified in our study are similar to those identified in previous studies(31, 32, 61-63). This included issues around environmental context and resources, therapist knowledge and the key role played by mentors and peers supporting previous research (49).
**Reviewer 2's comments**	
Thanks a lot for the opportunity you have offered me to revise the manuscript "Mixed methods non-randomised single-arm feasibility study assessing delivery of a remote vocational rehabilitation intervention for patients with serious injury: the ROWTATE study". I thank the authors for their efforts in producing this study.	We thank the reviewer for this positive comment.
As a significant strength, this paper evaluates therapists' training and competence, adapts the intervention and training for remote delivery and assesses feasibility and fidelity of remote delivery to inform a definitive randomised controlled trial.	We thank the reviewer for these positive comments.
As a weakness, the manuscript presents a few issues and needs more details and clarity concerning methodological elements, results presentation and discussion that are useful for understanding its content.	We have addressed the specific issues in the comments below.
GENERAL<br>*abbreviations: please report ALL the abbreviations in full in the whole manuscript.	We have replaced most of the abbreviations with the full term, including those for Vocational rehabilitation, Theoretical Domains Framework, Evidence-Based Practice Attitude Scale, Evidence Based Practice

	Confidence Scale, case report forms, major trauma centres, injury severity score, Patient and Public Involvement, Project Steering Committee, post-traumatic stress disorder, team objective structured clinical examination, National Health Systems, Microsoft Teams, Hospital Anxiety and Depression Scale -Anxiety, Impact of Event Scale-Revised, Montreal Cognitive Assessment score, Theoretical Framework of Acceptability, spinal cord injury, General Practitioner, return-to-work, Health and Care Professionals Council.<br><br>We have retained those for ROWTATE, OT and CP due to the frequency of their use in the manuscript. We have also kept the statical abbreviations (e.g. SD, IQR) and other terms (e.g., IT, COVID-19, CONSORT, Medline, EMBASE and PsycINFO, EQ-5D-5L) due to their conventional use.
*English: please revise the English of the manuscript	We have now made several revisions to the English language in the manuscript including the corrections suggested by reviewer one. All changes are highlighted in red font.
TABLES and FIGURES:<br>*abbreviations: please report ALL the abbreviations in full in a legend (also in the Supplementary file).	We have replaced most of the abbreviations with the full term, including those for Vocational rehabilitation, Theoretical Domains Framework, Evidence-Based Practice Attitude Scale, Evidence Based Practice Confidence Scale, case report forms, major trauma centres, injury severity score, Patient and Public Involvement, Project Steering Committee, post-traumatic stress disorder, team objective structured clinical examination, National Health Systems, Microsoft Teams, Hospital Anxiety and Depression Scale -Anxiety, Impact of Event Scale-Revised, Montreal Cognitive Assessment score, Theoretical Framework of Acceptability, spinal cord injury, General Practitioner, return-to-work, Health and Care Professionals Council.

	We have retained those for ROWTATE, OT and CP due to the frequency of their use in the manuscript. We have also kept the statical abbreviations (e.g. SD, IQR) and other terms (e.g., IT, COVID-19, CONSORT, Medline, EMBASE and PsycINFO, EQ-5D-5L) due to their conventional use.
*add a figure to synthesise the findings	We attempted to create a figure for the findings but this proved to be very difficult without using extensive text.
#ABSTRACT:<br>*keywords: please check if the keywords were MeSh terms.	We have now replaced all the keywords in the manuscript with MeSh terms. However, the BMJ open submission system only allows choice of key words from the submission system which is different form the MeSH terms.
#INTRODUCTION<br>* background and rationale: Please strengthen the value of remote training to promote education to healthcare providers. A useful reference could be this one (doi: 10.1007/s40670-021-01439-z).	Thank you for this suggestion. We have now added 3 studies to support the value of remote training. This now reads<br><br>*"Recent systematic reviews have identified adequate training to be essential to enhance competency in health professionals and overcome challenges of remote healthcare delivery[38], including evidence amongst OTs[39]. The importance of remote training for healthcare professionals has also been highlighted[40]."*
#METHODS and RESULTS:<br>*Reporting and analysis: it is a mixed methods, but there is no evidence of the use of frameworks shared by the scientific community (e.g., Creswell and Clark) that considers sequence, priority and integration of findings and their transformation gradient (QUANT-□QUAL or QUAL □ QUANT) [Creswell JW, Clark VL. Designing and conducting mixed methods research. 3rd ed. Thousand Oaks, CA, USA: Sage Publications, 2018].<br><br>In addition, the Good Reporting of a Mixed Methods Study (GRAMMS) criteria for mixed methods was not used [O'Cathain A, Murphy E, Nicholl J. The quality of mixed methods studies in health services research. J Health Serv Res Policy 2008; 13: 92-98]. Please revise and organize accordingly	Thank you for this suggestion. As different study objectives were met using different study designs we have opted in providing a separate for each description in Figure 1.<br><br>We have also provided more details in the analysis section on how we integrated the different types of methodologies. This now reads:<br>*Integration of methods*<br>*Integration of methods occurred during the data analysis and interpretation stages, which occurred concurrently. Our interpretations were borne from comparing and contrasting findings from each dataset for each objective. Finally, we used a narrative synthesis*

	approach, whereby we wove together the key findings from the different approaches, based on the Theoretical Domains Framework [53] and the Theoretical Framework of Acceptability.[62] Discrepancies or "unexpected" findings were discussed with the wider research team to make sense of the findings and why divergence occurred.<br><br>A GRAMMS checklist has been added to the tables in the manuscript (table 7). Changes were made to the description of mixed-methodology to complete the missing information as required by the GRAMMS checklist.
#DISCUSSION<br>*reporting: please organize the discussion as main findings, comparison with evidence, strengths and weakness, possible implications for clinical practice and research. I suggest adding the implications.	We have now updated the headings in the discussion to use the recommended one suggested by reviewer 2. The discussion finishes with a section on implication section relating to the main trial. We have also made reference to implications of findings to other similar trials.

VERSION 2 - REVIEW

Reviewer 2

Name Rossettini, Giacomo

Affiliation University of Genoa, Neuroscience, Rehabilitation, Ophthalmology, Genetics, Maternal and Child Health

Date 03-Oct-2025

COI

Dear Authors,

You did a great revision.

I am happy to accept it in this form.

Best regards.
